# Molecular understanding of calcium permeation through the open Orai channel

**DOI:** 10.1371/journal.pbio.3000096

**Published:** 2019-04-22

**Authors:** Xiaofen Liu, Guangyan Wu, Yi Yu, Xiaozhe Chen, Renci Ji, Jing Lu, Xin Li, Xing Zhang, Xue Yang, Yuequan Shen

**Affiliations:** 1 State Key Laboratory of Medicinal Chemical Biology and College of Life Sciences, Nankai University, Tianjin, China; 2 School of Medicine, Zhejiang University, Hangzhou, China; 3 Synergetic Innovation Center of Chemical Science and Engineering, Tianjin, China; Temple University, UNITED STATES

## Abstract

The Orai channel is characterized by voltage independence, low conductance, and high Ca^2+^ selectivity and plays an important role in Ca^2+^ influx through the plasma membrane (PM). How the channel is activated and promotes Ca^2+^ permeation is not well understood. Here, we report the crystal structure and cryo-electron microscopy (cryo-EM) reconstruction of a *Drosophila melanogaster* Orai (dOrai) mutant (P288L) channel that is constitutively active according to electrophysiology. The open state of the Orai channel showed a hexameric assembly in which 6 transmembrane 1 (TM1) helices in the center form the ion-conducting pore, and 6 TM4 helices in the periphery form extended long helices. Orai channel activation requires conformational transduction from TM4 to TM1 and eventually causes the basic section of TM1 to twist outward. The wider pore on the cytosolic side aggregates anions to increase the potential gradient across the membrane and thus facilitate Ca^2+^ permeation. The open-state structure of the Orai channel offers insights into channel assembly, channel activation, and Ca^2+^ permeation.

## Abstract 2

The structure of a constitutively active mutant of the fruit fly Orai calcium influx channel reveals a conformational transduction pathway upon channel activation and suggests an anion-assisted mechanism for calcium permeation.

## Introduction

Calcium signaling is essential in a broad range of biological processes [1]. In metazoans, store-operated calcium entry (SOCE) is one of the major extracellular calcium influx pathways not only in excitable cells but also particularly in nonexcitable cells [2,3]. The action of extracellular ligands triggers the release of Ca^2+^ from the endoplasmic reticulum (ER). The stromal interaction molecule (STIM) located on the ER senses Ca^2+^ depletion within the ER and, in response, undergoes oligomerization and translocation to the ER–plasma membrane (PM) junction, where it couples with and activates the Ca^2+^-selective channel Orai [4,5]. The Orai protein belongs to the family called store-operated calcium channels (SOCs), which have unique features among ion channels, including voltage independence, low conductance, and high Ca^2+^ selectivity [6–8]. In humans, there are 3 isoforms (Orai1–3) of Orai proteins. They are highly homologous to each other (approximately 62% overall sequence identity) [5]. Each Orai contains 4 transmembrane (TM) helices with the N-terminal and C-terminal ends located inside the cytosol [9]. Orai channel–mediated Ca^2+^ signaling plays an important role in multiple physiological processes [10]. Consequently, loss-of-function mutations and gain-of-function mutations of Orai1 have been identified in human patients and found to cause various diseases, such as severe combined immunodeficiency, skeletal myopathy, Stormorken syndrome, and others [11,12].

The Orai channel is assembled from multiple subunits [13,14]. The precise stoichiometry of the Orai channel has been in great debate. Several groups have reported that the Orai channel is a dimer in the resting state and forms a tetramer in the activated state [15,16]. The crystal structure of *Drosophila* Orai showed a trimer of the Orai dimer, forming an approximately hexameric assembly [17]. However, the hexameric stoichiometry of the Orai channel was brought into question by studies of single-molecule photobleaching [18] and artificially linked hexameric concatemers of human Orai (hOrai) 1 [19]. Recent studies of the concatenated Orai1 channel indicated that the Orai1 channel functions as a hexamer [20–22]. Therefore, hexameric stoichiometry is generally accepted as one of the major conformations of the Orai channel [5,23,24].

The current crystal structure of *Drosophila melanogaster* hexameric Orai represents an inactive conformation [17]. The innermost TM1 from each subunit forms a closed ion pore, and 3 other TM helices are arranged around TM1. The selectivity filter is presumably formed by a ring of 6 glutamate residues on the extracellular side of the pore. The mechanism of channel activation and Ca^2+^ permeation remains unclear. Here, we determined the structure of the constitutively active *Drosophila melanogaster* Orai (dOrai) mutant P288L using both X-ray crystallography and cryo-electron microscopy (cryo-EM). The open state of the dOrai structure depicts the mechanism of channel activation and Ca^2+^ permeation.

## Results

### The dOrai-P288L channel is constitutively active

**Fig 1:**
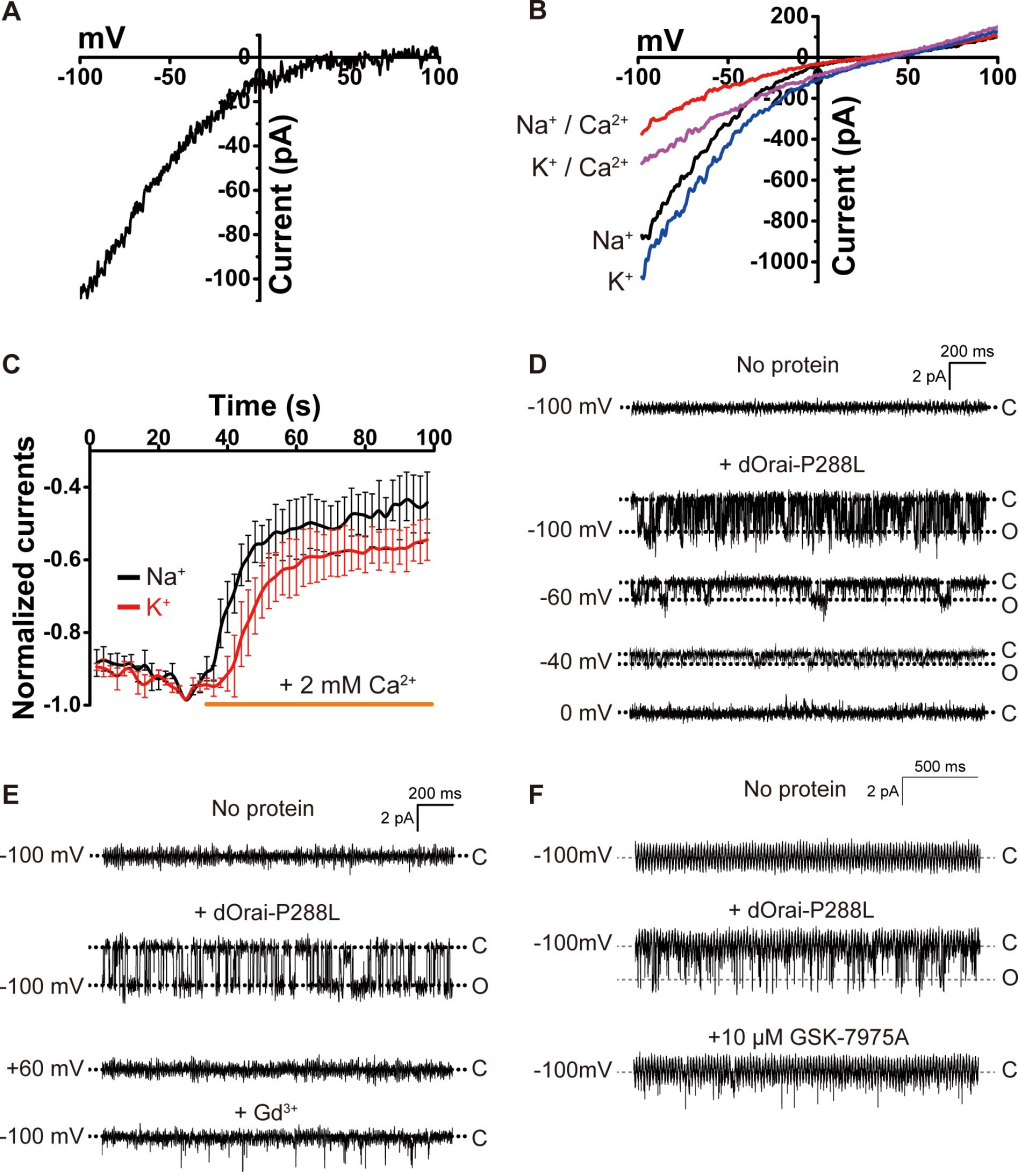
The dOrai-P288L channel is constitutively active. (A) Current-potential (I–V) trace of the whole-cell Ca^2+^ current induced by the ramp potential. At least 3 independent experiments were conducted. (B) Representative I–V curves of the whole-cell Na^+^ and K^+^ currents induced by the ramp potential with external Na^+^ and K^+^ recording solutions before and after the addition of 2 mM Ca^2+^. (C) Representative time course of the normalized Na^+^ and K^+^ currents without or with the addition of 2 mM Ca^2+^. Data are shown as the mean ± SEM (*n* = 4 independent experiments). (D) Single-channel Ca^2+^ currents evoked by the indicated stimulation voltages before and after the addition of purified proteins. (E) The effect of 20 μM Gd^3+^ on the single-channel Ca^2+^ current. (F) The effect of 10 μM GSK-7975A on the single-channel Ca^2+^ current. Primary data can be found in S1 Data. dOrai, *Drosophila melanogaster* Orai; GSK, GlaxoSmithKline.

To obtain the three-dimensional structure of constitutively active Orai, a construct of dOrai (hereafter referred to as dOrai-P288L) consisting of amino acids 132 to 341 containing 3 mutations (C224S, C283T, and P288L) was selected and purified (S1 Fig). The dOrai P288L mutant corresponds to the P245L gain-of-function mutant of hOrai1, which causes the overlapping syndromes of tubular myopathy and congenital miosis [12,25]. To verify that the dOrai-P288L channel was constitutively active, electrophysiology was carried out. First, we transiently transfected human embryonic kidney (HEK)-293T cells with a plasmid encoding the green fluorescent protein (GFP)-tagged dOrai-P288L channel and showed that the channel was localized on the cell surface (S2A Fig). Next, we used whole-cell patch clamp measurements to record the Ca^2+^ current in HEK-293T cells expressing the dOrai-P288L channel and found that the inward-rectified Ca^2+^ current had a reversal potential of 36.9 ± 1.4 mV (Fig 1A). Furthermore, in the absence of divalent cations, we observed clear monovalent cation currents (Fig 1B). The addition of Ca^2+^ significantly inhibited the monovalent cation currents (Fig 1B and 1C). Finally, we used single-channel current recording in vitro to verify the activity of the purified dOrai-P288L channel. The monodisperse and high-purity protein (S2B Fig) was reconstituted into a planar lipid bilayer, as reported by our previous research [26]. No current signal was observed at −100 mV without addition of the channel protein. After addition of the channel protein, obvious inward-rectified Ca^2+^ currents of approximately 2 pA at −100 mV were recorded (Fig 1D). The single-channel Ca^2+^ currents gradually diminished as the stimulation potential depolarized from −100 mV to 0 mV. Additionally, the single-channel Ca^2+^ currents of the dOrai-P288L channel were significantly inhibited by Gd^3+^ (Fig 1E) or the specific Orai channel inhibitor GlaxoSmithKline (GSK)-7975A (Fig 1F). Taken together, these results show that the dOrai-P288L channel is fully active and recapitulates the properties of the STIM-activated Orai channel.

### Overall structure of the open channel

**Fig 2:**
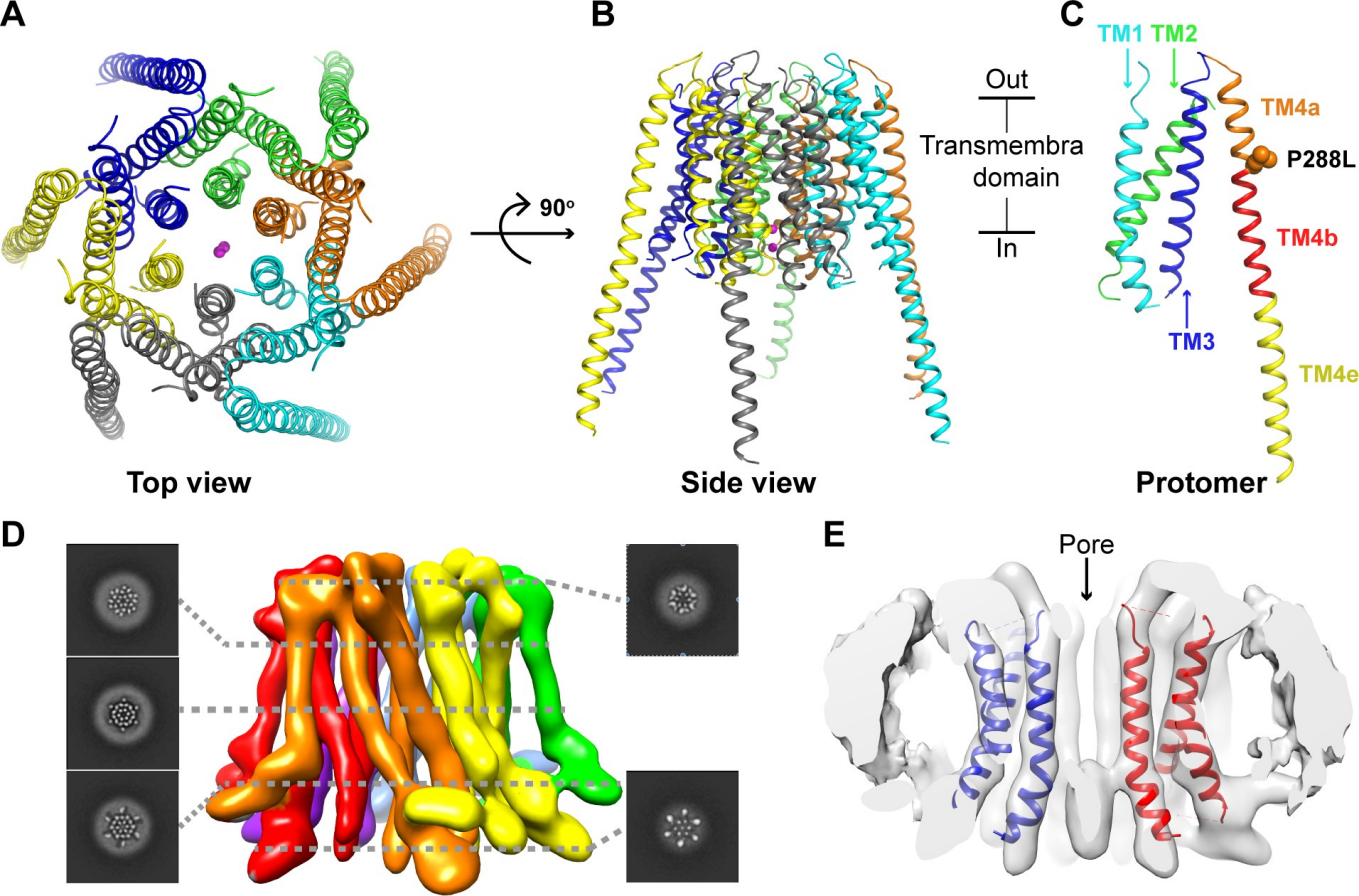
Overall structure of the open state of the dOrai channel. (A) Top view of the structure of the open dOrai channel. The 6 protomers are colored green, blue, yellow, gray, cyan, and orange. Two ions are shown as spheres in the center and are colored magenta. (B) Side view of the structure of the open dOrai channel. (C) The architecture of 1 protomer. The TM1, TM2, and TM3 helices are colored cyan, green, and blue, respectively. The TM4 helices are further divided into 3 sections, labeled TM4a (orange), TM4b (red), and TM4e (yellow). The side chains of the mutated residue P288L are shown as orange spheres. (D) Side view of the final three-dimensional reconstruction of the open dOrai channel with slices of the indicated levels. Each protomer is colored individually. (E) Overlay of the cryo-EM map (white surface) with the crystal structure model (color cartoon) of the open dOrai channel. The 2 protomers are colored blue and red, respectively. cryo-EM, cryo-electron microscopy; dOrai, *Drosophila melanogaster* Orai; TM, transmembrane.

The crystal structure of the dOrai-P288L channel was determined at a resolution of 4.5 Å by molecular replacement using the closed dOrai structure (Research Collaboratory For Structural Bioinformatics [RCSB] code: 4HKR) as a model. The overall architecture of the open channel indicates a hexameric assembly (Fig 2A and 2B). Each protomer consists of 4 TM helices (TM1–TM4; Fig 2C). The 6 protomers adopt a 6-fold noncrystallographically symmetric arrangement, resulting in the 6 TM1 helices forming the ion-conducting pore in the center. The TM2 and TM3 helices from each protomer surround the 6 TM1 helices, forming a fence that fixes the position of the TM1 helices. Each TM4 helix forms an extralong helix, extending into the cytosol. There are 2 hexamers in each asymmetric unit of the dOrai-P288L crystal that are bound together through coiled-coil interactions between the TM4 helices (S3 Fig).

To further confirm the open-state conformation of the dOrai-P288L channel, we used cryo-EM methodology. Negative-stain electron microscopy was used to screen different conditions, including the presence of amphipols (A8–35) and detergents (n-dodecyl-β-d-maltopyranoside [DDM]) as well as reconstitution into nanodiscs. Negatively stained dOrai-P288L channels in the nanodiscs appeared to be monodisperse and were further subjected to cryo analysis. The samples had an orientation bias, with more than 95% oriented to the same top or bottom view. We overcame this problem by using detergent glyco-diosgenin (GDN). Because the dOrai-P288L channel was wrapped in a thick layer of detergents, the final structure was determined at an overall resolution of 5.7 Å by single-particle cryo-EM (S4 and S5 Figs). Each helix (TM1, TM2, TM3, and TM4) was well resolved in the final density map (Fig 2D). The overall cryo-EM structure of the dOrai-P288L channel was similar to its crystal structure counterpart. The crystallographic dimer across the central pore fit well in the cross-section of the cryo-EM density map (Fig 2E).

### Activation of the Orai channel

**Fig 3:**
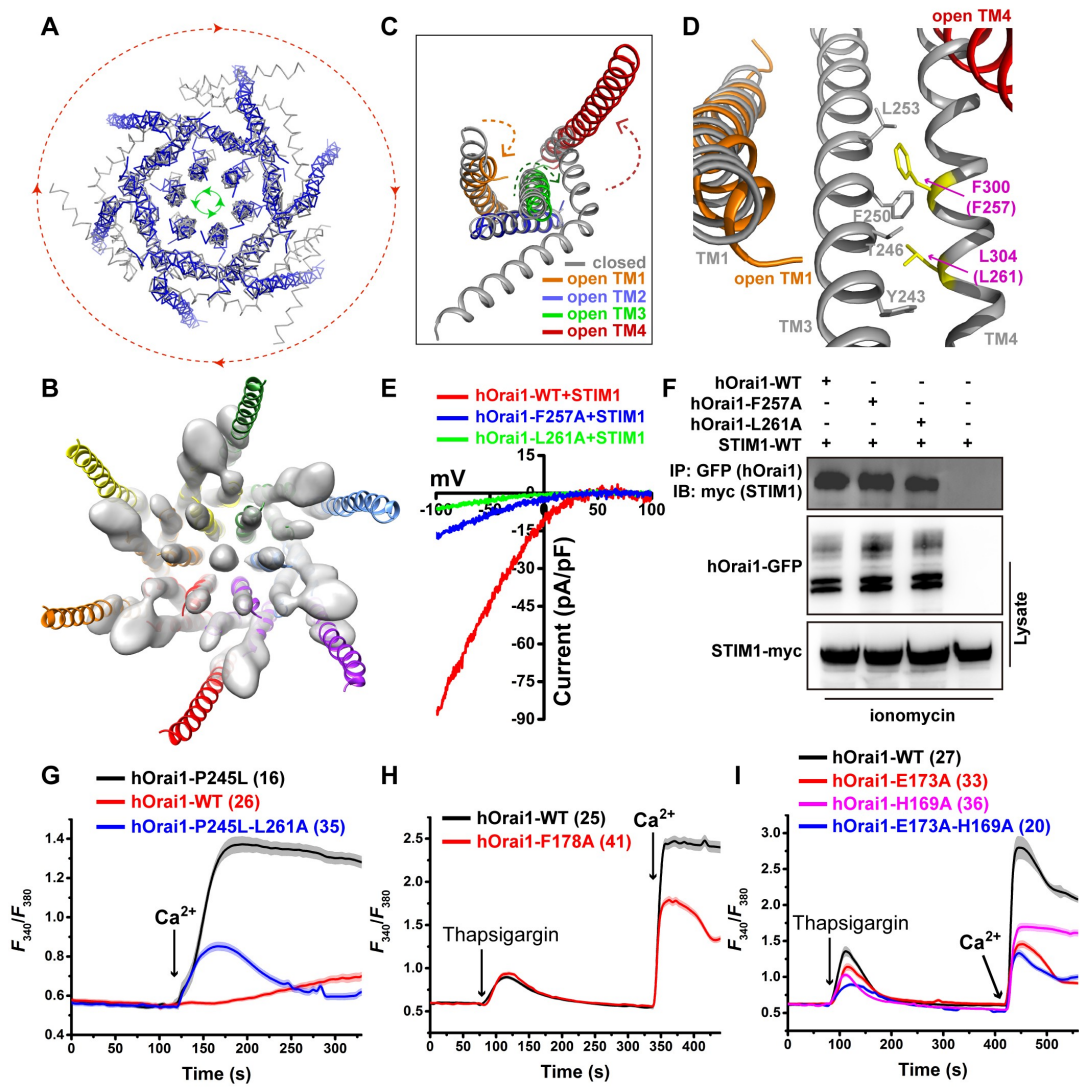
Conformation of the transduction pathway during channel opening. (A) Top view of the overlay of closed (gray) and open (blue) dOrai channels. During opening, the 6 peripheral TM4 helices rotate clockwise (red arrow direction), while the 6 innermost TM1 helices on the cytosolic side rotate counterclockwise (green arrow direction). (B) Bottom view of the overlay of the open dOrai channel between the crystal structure (color cartoon) and the cryo-EM density map (white surface). The density in the middle presumably represents anions. (C) Overlay of 1 protomer between the closed (gray) and open (color) states of the dOrai channel. Arrows denote possible helix movement during opening. (D) Interactions between TM3 and TM4 in the closed state. Two residues, F300 and L304, are colored yellow. The residue number in hOrai1 is shown in parenthesis. (E) Representative I–V curves of the whole-cell Ca^2+^ currents of STIM1-activated wild-type hOrai1 and mutants. (F) Western blot analysis of hOrai1-GFP (wild type and mutants) coimmunoprecipitated with STIM1-myc. (G–I) Extracellular Ca^2+^ influx in HEK-293T cells expressing hOrai1-GFP (wild type and mutants). The number of analyzed cells is indicated. Error bars denote the SEM. Primary data can be found in S1 Data. cryo-EM, cryo-electron microscopy; dOrai, *Drosophila melanogaster* Orai; GFP, green fluorescent protein; HEK, human embryonic kidney; hOrai1, human Orai1; IB, immunoblot; IP, Immunoprecipitation; STIM, stromal interaction molecule; TM, transmembrane; WT, wild type.

Structural comparison of the open and closed dOrai structures revealed that the TM1 to TM3 regions have a similar architecture, whereas the TM4 helices are completely different (Fig 3A). From the top view, all 6 TM4 helices are fully extended, and a clockwise rotation occurred during opening, causing the N-terminal regions of the innermost 6 TM1 helices to twist outward in a counterclockwise direction. These conformational changes are also observed in the cryo-EM density map of the dOrai-P288L channel (Fig 3B). Of note, the TM4e segment of the TM4 helix is invisible in the cryo-EM density map. By contrast, the closed conformation of the dOrai channel does not fit into our cryo-EM density map (S6 Fig) as well the open conformation according to the correlation coefficient (0.562 for the open conformation versus 0.459 for the closed conformation) calculated by the Phenix program [27]. The twisted region starts from the positively charged residue K159, which corresponds to residue K87 in hOrai1, and proceeds to the N-terminus of the TM1 helix (Fig 3C and S7 Fig). We were not able to distinguish whether the remaining region of the TM1 helix rotated [23] during opening in either our crystallographic structure or the cryo-EM density map.

Within each protomer, we observed a conformational transduction pathway from the peripheral TM4 helix through the middle TM3 helix to the basic section of the innermost TM1 helix (Fig 3C). To confirm that such conformational transduction occurs during opening, we made 2 mutants (hOrai1-F257A and hOrai1-L261A) of wild-type hOrai1. Two residues, F257 and L261, in hOrai1 correspond to F300 and L304 in dOrai, respectively, which form hydrophobic interactions between the TM3 helix and TM4 helix in the closed conformation of dOrai (Fig 3D) [28]. Upon opening, the drastic swing of the TM4b portion presumably causes the movement of the TM3 helix (Fig 3C). As expected, whole-cell patch clamp measurements showed that the Ca^2+^ currents of hOrai1-F257A and hOrai1-L261A after activation by STIM1 were significantly lower than those of wild type (Fig 3E and S8A Fig) [28]. We also used a Ca^2+^ influx assay to cross-validate this result. Two mutants (hOrai1-F257A and hOrai1-L261A) completely lost the extracellular Ca^2+^ influx after the Ca^2+^ in the ER was depleted (S8B Fig). To ensure that the reduced channel activity was indeed caused by the reduction in channel activation rather than attenuated STIM1-Orai1 binding, we performed coimmunoprecipitation (co-IP) and intracellular fluorescence resonance energy transfer (FRET) experiments to verify the interaction between the hOrai1 mutations and STIM1. When co-expressed with STIM1-myc, wild-type and mutant hOrai1-GFP were similarly able to pull down STIM1 after ionomycin treatment (Fig 3F). Additionally, when yellow fluorescent protein (YFP)-tagged STIM1 and cyan fluorescent protein (CFP)-tagged wild-type and mutant hOrai1 were co-transfected into HEK-293T cells, the measured apparent FRET efficiency (Eapp) values after the cells were treated with thapsigargin were similar (S8C Fig). Moreover, we made an L261A mutation into the constitutively active mutant hOrai1-P245L to create the mutant hOrai1-P245L-L261A and carried out an extracellular Ca^2+^ influx assay. Compared with the mutant hOrai1-P245L, the mutant hOrai1-P245L-L261A showed much less extracellular Ca^2+^ influx (Fig 3G). We also made the mutant hOrai1-F178A (F250 in dOrai), which contained a mutation in the TM3 helix, and this mutant showed significantly attenuated extracellular Ca^2+^ influx (Fig 3H). These results indicate that after interference of the interaction between the TM3 helix and the TM4 helix, hOrai1 cannot be activated by STIM1 but is able to normally associate with STIM1, consistent with previously published results [28].

Furthermore, in the closed conformation of dOrai, 3 residues—L153, S154, and K157, which correspond to L81, S82, and K85 in hOrai1, respectively—were shown to take part in the interaction between the basic section of the TM1 helix and the TM3 helix (S9 Fig). We then made 3 mutants (hOrai1-E173A, hOrai1-H169A, and hOrai1-E173A-H169A) in the TM3 helix with the aim of interfering with the interactions between the TM1 helix and the TM3 helix. Comparing to the wild type, these 3 mutants obviously decreased the extracellular Ca^2+^ influx (Fig 3I). Moreover, earlier reports showed that the hOrai1-L81A mutant completely blocked the function of the channel without altering the STIM1-Orai1 association, and furthermore, the triple L81A-S82A-K85E mutant or the double L81A-S82A mutant prevented extracellular Ca^2+^ influx via the constitutively active hOrai1-ANSGA mutant channel [28]. The single-replacement K85E in hOrai1 resulted in a complete absence of the STIM-dependent current in cells in response to Ca^2+^ store depletion [29]. Taken together, these data show that the conformational transduction pathway (T4b helix → T3 helix → T1 helix basic section) identified in our open-state structure is critical for Orai channel activation and also provides structural evidence for the previously postulated gating mechanism of hOrai1 by STIM1 [28].

### Mechanism of Ca^2+^ permeation

**Fig 4:**
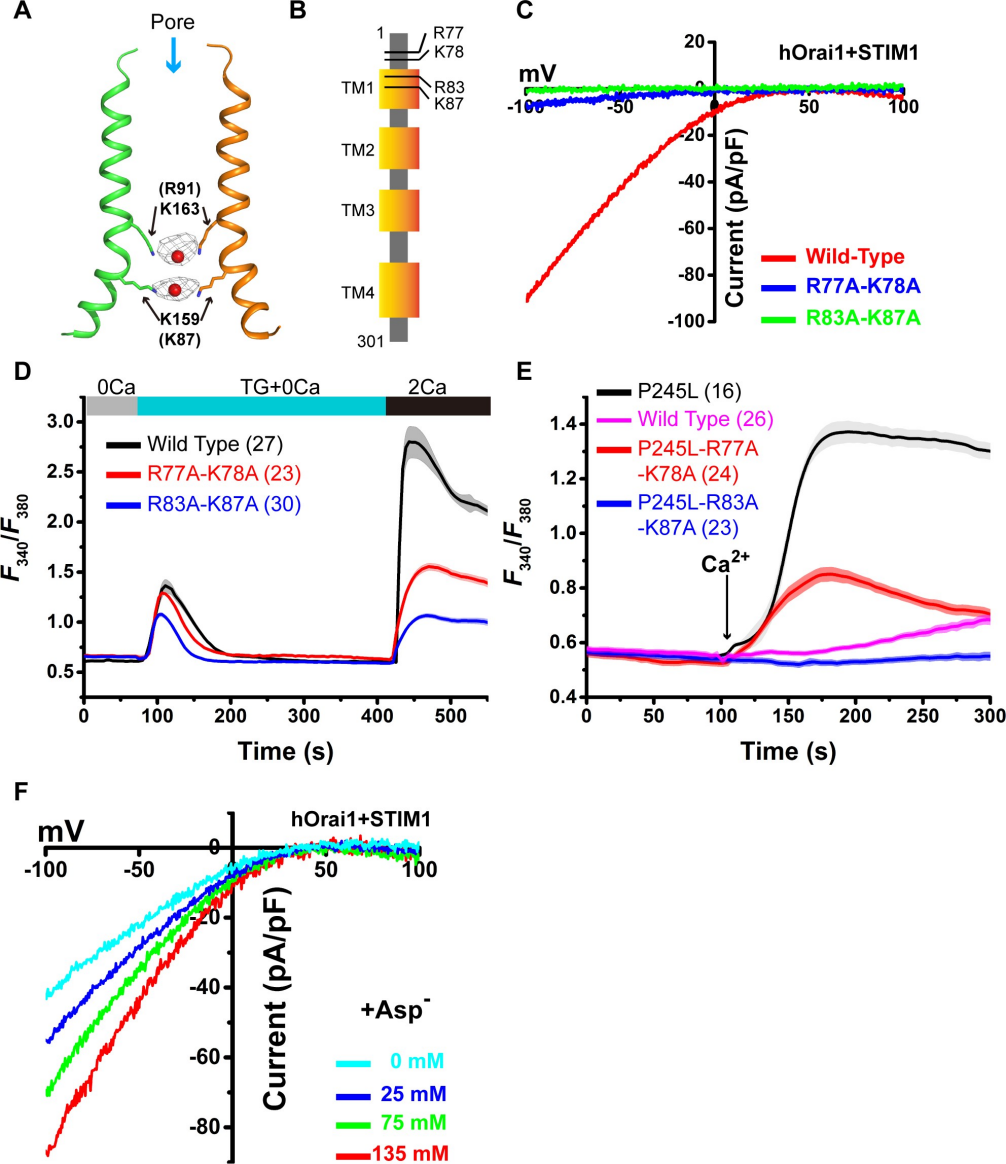
Anions facilitate Ca^2+^ permeation. (A) Anion binding in the pore. The 2***F***o-***F***c Fourier electron density map contoured at 2.0 σ for anions is shown as a gray mesh. TM1 helices from 2 opposite subunits are depicted. Residues K163 and K159 are shown as sticks, and anions are shown as red spheres. (B) Schematic model of the hOrai1 channel. The positions of 4 basic residues (R77, K78, R83, and K87) are indicated. (C) Representative I–V curves of the whole-cell Ca^2+^ currents of STIM1-activated wild-type hOrai1 and mutants. (D) Extracellular Ca^2+^ influx in HEK-293T cells co-expressing STIM1-YFP and wild-type or mutant hOrai1-GFP. (E) Extracellular Ca^2+^ influx in HEK-293T cells expressing wild-type or mutant hOrai1-GFP. (F) Representative I–V curves of the whole-cell Ca^2+^ currents of STIM1-activated wild-type hOrai1 at the indicated concentration of cesium aspartate. Primary data can be found in S1 Data. Asp, aspartate; GFP, green fluorescent protein; HEK, human embryonic kidney; hOrai1, human Orai1; STIM, stromal interaction molecule; TM, transmembrane; YFP, yellow fluorescent protein.

In the closed state of the dOrai structure, the basic section of the TM1 helix was identified and proposed to provide electrostatic repulsion in blocking cation transport [17,30]. During Ca^2+^ permeation, this positively charged region must be neutralized or shielded to avoid electrostatic repulsion. If this argument was true, mutations in the basic section of the TM1 helix would significantly enhance Ca^2+^ permeation through the open Orai channel. However, several published results have shown that the basic section of the TM1 helix is indispensable for Ca^2+^ permeation in the Orai channel [25,28,31]. In our open-state dOrai structure, electron density peaks corresponding to anions can be found around the basic section of the TM1 helix in both the crystallographic structure (Fig 4A) and the cryo-EM model (Fig 3B). A similar anion binding site has also been reported in the crystal structure of the closed state of dOrai [17]. These results suggest that the binding of these anions to the basic section of the TM1 helix may be necessary for Ca^2+^ permeation during channel opening. With this possibility in mind, mutating these basic amino acids will reduce the binding of anions, leading to an attenuated Orai channel and thus reconciling the above functional data. By contrast, introducing more anions on the cytosolic side of the membrane may potentiate Ca^2+^ permeation through activated Orai channels.

Indeed, 2 mutants (hOrai1-R83A-K87A and hOrai1-R77A-K78A) showed severely attenuated Ca^2+^ currents in the STIM1-activated hOrai1 channel by whole-cell patch clamp measurements (Fig 4B and 4C and S10A Fig) and significantly decreased store-depleted extracellular Ca^2+^ influx (Fig 4D). The reduced channel activity was not caused by attenuated STIM1-Orai1 binding because the hOrai1 mutants pulled down STIM1 at a similar level to wild-type hOrai after ionomycin treatment in a co-IP experiment (S10B Fig). Moreover, we made R77A, K278A, R83A, and K87A mutations into the channel of the constitutively active mutant hOrai1-P245L to create 2 mutants (P245L-R77A-K78A and P245L-R83A-K87A) and carried out the extracellular Ca^2+^ influx assay. Compared with the mutant P245L, the 2 mutants showed much less extracellular Ca^2+^ influx (Fig 4E). These results reinforce the argument that the basic amino acids in this region are important for Ca^2+^ permeation in the Orai channel. We added a certain amount of aspartate in the pipette solution to record the Ca^2+^ current in the STIM1-activated hOrai1 channel. As expected, aspartate concentration-dependent Ca^2+^ currents were observed (Fig 4F and S10C Fig). These results suggest that the basic section of the TM1 helix may aggregate negative charges to facilitate Ca^2+^ permeation during the opening of the Orai channel.

## Discussion

**Fig 5:**
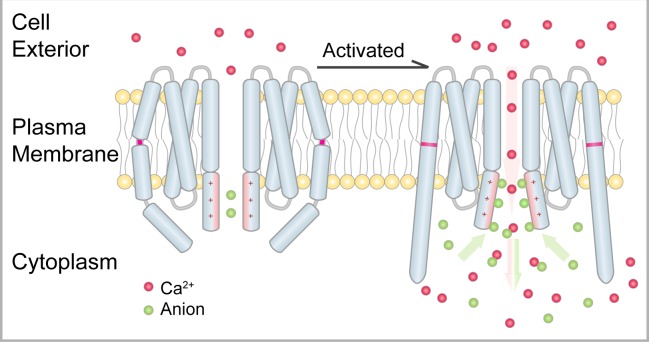
Proposed mechanism of Orai channel activation and Ca^2+^ permeation. Two opposite subunits of the Orai channel are shown. Ca^2+^ and anions are drawn as red and green spheres, respectively. In the closed state, the latched TM4 helix limits the opening of the pore on the cytosolic side. Upon activation, the swing of the TM4 helix induces an outward twist of the pore on the cytosolic side, thus aggregating anions. These anions may reduce positive charge repulsion and increase the potential gradient across the membrane, allowing Ca^2+^ permeation. TM, transmembrane.

In this study, we determined the structure of the constitutively active dOrai-P288L channel mutant by X-ray crystallography and cryo-EM reconstruction. The open state of the dOrai channel had a hexameric assembly. Structural comparison between the open state and the closed state revealed a conformational transduction pathway from the peripheral TM4 helix to the innermost TM1 helix. Mutations that interfered with the pathway dramatically attenuated the STIM1-activated Orai function, as demonstrated by electrophysiology. Twisting of the basic section of the TM1 helix to face the cytosolic side may accommodate more anions to facilitate Ca^2+^ permeation. Therefore, we propose a model of Ca^2+^ permeation in the Orai channel (Fig 5). In the closed state of the channel, the latched TM4 helix closes the pore on the cytosolic side. Positive charge repulsion and anion plugs block Ca^2+^ permeation. Upon opening, the TM4 helix swing twists the basic section outward to accommodate more anions. These anions not only neutralize the positive charges to reduce charge repulsion but also increase the potential gradient across the membrane, thus facilitating Ca^2+^ permeation.

This model is consistent with many published functional studies. Zhou and colleagues identified a “nexus” site (amino acids 261–265) within the hOrai1 channel that is proposed to connect the peripheral C-terminal STIM1-binding site to the hOrai1 pore helices [28]. Structural comparison of the dOrai channel structures between our open state and the published closed state clearly indicated a conformational transduction pathway (T4b helix → T3 helix → T1 helix basic section), providing further evidence that the “nexus” site is most likely the trigger for channel activation. Furthermore, cholesterol has been reported to interact with the hOrai1 channel and inhibit its activity through residues hOrai1-L74 and hOrai1-Y80 [32]. These 2 residues are located within the interface between the TM1 helix and the TM3 helix. Cholesterol binding presumably interrupts the conformational transduction pathway, which explains why cholesterol did not affect the binding of STIM1 to the hOrai1 channel but attenuated hOrai1 activation [32].

How Orai channels conduct Ca^2+^ is a puzzling question. The Orai pore consists of an extracellular mouth, a selectivity filter, an unusually long hydrophobic cavity, and an intracellular basic region. The dOrai closed-state structure shows that the narrowest region of the pore has a diameter of 6.0 Å, wide enough for a dehydrated Ca^2+^ to pass through. Yamashita and colleagues proposed rotating the pore helix upon channel activation [23]. However, Frischauf and colleagues reported that they did not observe any rotation of the pore helix in molecular dynamic simulation studies [33]. In our open-state structure of the dOrai channel, rotation of the pore helix was not observed. Recently, another crystal structure of the open state (H206A) of the dOrai channel at low resolution (6.7 Å) was reported [34]. Hou and colleagues did not observe pore helix rotation either. Therefore, the mechanism of pore helix rotation is inconsistent with the results of structural studies.

Hou and colleagues proposed another pore-dilation model based on the structural findings of a dilated hydrophobic cavity and a wide open intracellular basic region [34]. Frischauf and colleagues reported that mutations in the TM2 helix may slightly increase the pore size in hydrophobic regions [33]. However, our structure showed the opening of the intracellular basic region but not the dilated hydrophobic cavity. Moreover, the pore-dilation model is inconsistent with the result that mutating the intracellular basic region of constitutively active hOrai1 abolished the channel activity (Fig 4E) [28]. Therefore, the pore-dilation mechanism is less likely to account for Ca^2+^ permeation of the Orai channel.

Our proposed anion-assisted Ca^2+^ permeation model is reasonable for explaining these results. Furthermore, it has been reported that Orai currents are inhibited by acidic but potentiated by basic intracellular solutions in various cell types [35]. This result is consistent with our model because basic intracellular solutions provide more hydroxide anions, whereas acidic solutions provide more proton cations. Both the pore helix rotation and pore-dilation models are difficult to explain. In summary, our studies provide a reasonable model that clarifies the molecular details of the activation and Ca^2+^ permeation of the Orai channel.

Of note, the single-channel Ca^2+^ conductance of the dOrai-P288L channel in our study is approximately 20 pS (Fig 1D), which has a 40-fold greater unitary Ca^2+^ conductance (approximately 0.5 pS) than a mutant hOrai1 channel [22]. We think that the discrepancy may be due to the different methodology used to measure single-channel conductance. The 2 channels differ because of the different species (*H*. *sapiens* versus *D*. *melanogaster*), different mutations (wild type versus P288L), and different experimental systems used (perforated whole-cell recording with noise analysis versus purified protein in lipid biolayer). Nevertheless, in our study, the single channel Ca^2+^ conductance of the dOrai-P288L channel is inward rectified, inhibited by Gd^3+^ and by the chemical GSK-7975A, which is a specific Orai channel blocker (Fig 1D–1F), thus recapitulating the properties of the STIM-activated Orai channel.

## Materials and methods

### Plasmid construct

The human full-length Orai1 and STIM1 were inserted into the pEGFP-N1, pEYFP-N1, pECFP-N1, and pCDNA3.1/myc-His to generate hOrai1-GFP, STIM1-YFP, hOrai1-CFP, and STIM1-Myc. *D*. *melanogaster* full-length Orai (Uniprot:Q9U6B8) codons were optimized for *Homo sapiens* and synthesized in Genewiz (Suzhou, China). To improve the expression and stability of target protein, we chose a fragment that contains amino acid residues 132 to 341 and introduced 2 cysteine mutations (C224S and C283T). Also, a P288L mutation was introduced to make an activated dOrai channel. This Orai segment (dOrai-P288L), containing 3 point mutations (C224S, C283T, and P288L), was cloned into a pEG-BacMam vector followed by a PreScission protease cleavage site and an enhanced green fluorescent protein (eGFP) at the C-terminus. All mutations were introduced using the Quick-Change Lightning Site-Directed Mutagenesis Kit (Agilent, Santa Clara, USA).

### Protein expression and purification

The dOrai-P288L construct was transformed into DH10Bac *Escherichia coli* cells to generate the bacmid. The bacmid was transfected into Sf9 cells to generate P1 baculovirus that were harvested after 72 h. P1 baculovirus were amplified in *Spodoptera frugiperda* (Sf) 9 cells to generate P2 baculovirus. P2 baculovirus were added (1%–3% v/v) to HEK293S-GnTi^−^ cells when the cells reached a density of 1.5–2.0 × 10^6^ cells/mL. To increase the expression level, 10 mM sodium butyrate was supplemented, and culture temperature was turned down to 30°C for 12 h post transduction, then further cultured for 60 h before harvesting. One liter of cells were resuspended and lysed by sonicator in 100 mL buffer A (20 mM Tris-HCl [pH 8.0], 200 mM NaCl, and 1 mM PMSF). The membrane fraction was collected by centrifugation at 180,000*g* in 4°C for 1 h. The membrane was then solubilized in 70 mL buffer A plus 1% n-dodecyl-β-d-maltopyranoside (DDM, Anatrace, Maumee), 0.2% cholesteryl hemisuccinate (CHS; Sigma, St. Louis), and 1× protease inhibitor cocktail (Roche, Indianapolis) with gentle shaking in 4°C for 1 h. Solubilized membranes were cleared by centrifugation at 100,000*g* for 30 min. Afterwards, supernatant was mixed with 3 ml Ni-NTA beads (GE Healthcare, Chicago) and incubated for 1 h. The beads were collected by a gravity column and washed by buffer B (20 mM Tris-HCl [pH 8.0], 200 mM NaCl, 0.03% DDM, 0.006% CHS) and buffer C (20 mM Tris-HCl [pH 8.0], 800 mM NaCl, 0.03% DDM, 0.006% CHS). Then, beads were mixed with 3C protease (1:50 v/v) to cleave eGFP tag overnight. The flow through was collected, and beads were washed 2 times by buffer B. All proteins were pooled and concentrated for further purification.

For crystal screen and optimization, the protein was injected into Superdex 200 Increase 10/300 GL (GE Healthcare, Chicago, USA) equilibrated by buffer D containing 20 mM Tris-HCl (pH 8.0), 80 mM NaCl, 2 mM DTT, 0.2% n-Nonyl-β-D-glucopyranoside (NG, Anatrace, Maumee), 0.28% n-Nonyl-β-D-Maltoside (NM, Anatrace, Maumee), and 0.1 mg/ml lipid (POPC:POPE:POPG = 3:1:1 [w/w]; Avanti Polar Lipids Inc, Alabaster); all peak fractions were collected and concentrated to 12 mg/mL.

For EM studies, the protein was loaded into Superdex 200 Increase 10/300 GL (GE Healthcare, Chicago) equilibrated by buffer E composed of 20 mM Tris-HCl (pH 8.0), 150 mM NaCl, 2 mM DTT, and 0.04% GDN (Anatrace, Maumee); the peak was concentrated to 0.8 mg/mL for negative-stain EM and cryo-EM grid preparation.

For the single-channel recording experiment, the protein was further purified using Superdex 200 Increase 10/300 GL (GE Healthcare, Chicago) equilibrated with buffer F (20 mM Tris-HCl [pH 8.0], 150 mM NaCl, 2 mM DTT, 0.02% DDM, and 0.004% CHS). Then peak fractions were collected and concentrated.

### Crystallization and structure determination

The protein was mixed with reservoir solution at the ratio of 1:1 by sitting drop. The reservoir solution consists of 0.05 M NaCl, 0.02 M MgCl_2_, 0.1 M sodium citrate (pH 6.5), 16% to 20% PEG400. The crystal was grown in 3 to 4 d at 17°C. Crystal was cryoprotected in a solution containing 0.05 M NaCl, 0.02 M MgCl_2_, 0.1 M sodium citrate (pH 6.5), 45% PEG400, 0.4% NG, 0.56% NM, and 0.2 mg/mL lipid (POPC:POPE:POPG = 3:1:1) and was flash-frozen in liquid nitrogen.

The crystal structure of the dOrai-P288L channel was determined by molecular replacement; the closed state of the dOrai model was used as a search model in PHASER [36]. The structure refinement was performed with the PHENIX program [27], and re-iterated model building was performed manually in COOT [37]. The final refinement yielded an R_crystal_ value of 32.57% and an R_free_ value of 38.83%. Structure validation was performed using MOLPROBITY [38]. Crystallographic data and refinement statistics are reported in S1 Text.

### Nanodisc reconstitution

Membrane scaffold protein MSP1E3D1 was expressed and purified, and dOrai was reconstituted into nanodisc as previously described with modifications [39]. Briefly, lipids (POPC:POPG:POPE = 3:1:1) were dried under argon stream for 2 h and placed in the vacuum chamber overnight to remove residual chloroform. Lipids were resuspended by sonication in buffer containing 20 mM Tris-HCl (pH 8.0), 150 mM NaCl, 2 mM DTT, and 20 mM DDM to final lipid concentration 10 mM. dOrai-P288L, MSP1E3D1, and lipids were mixed at a molar ratio of 1:4:140. The mixture was incubated on ice for 1 h. Afterwards, Bio-beads (20 mg/mL; Bio-Rad) were added to the mixture and gently shaken at 4°C to start the reconstitution. After 3 h, Bio-beads were exchanged and then rotated overnight. The supernatant was loaded into Superdex 200 Increase 10/300 GL equilibrated with Gel-filtration buffer (20 mM Tris-HCl [pH 8.0], 150 mM NaCl, 2 mM DTT), and the peak fraction was collected and concentrated to 0.8 mg/mL for negative-stain EM and cryo-EM grid preparation.

### EM grid preparation and data collection

For cryo-EM sample preparation, an aliquot of 3.5 μl fresh sample of concentration 0.8 mg/ml was applied to a glow-discharged holy carbon grid (Quantifoil, R2/1, 200 mesh). The grids were blotted for 5 s under 100% humidity at 20°C and plunged into liquid ethane cooled by liquid nitrogen with an FEI Vitrobot Mark IV. The grid was loaded onto an FEI Titan Krios electron microscope with a K2 Summit direct electron counting detector (Gatan). Movies were collected by SerialEM (http://bio3d.colorado.edu/SerialEM/) with an under-focus range of 1.5 to 2.5 μm and under a magnification of 29,000× in super-resolution mode, corresponding to a pixel size of 0.507 A° on the specimen level. Each movie was dose fractionated to 40 frames, with a 0.2 s exposure time per frame and a dose rate of 1.6 counts per physical pixel per second, resulting in a total dose of 49.8 e^−^/ A°^2^. A total of 5,182 movies were acquired from 3 grids in an image session of 120 h.

### Cryo-EM image processing

The recorded movies were processed by MotionCorr2 [40] for a 5 × 5 patches drift correction with dose weighting and binned 2-fold, resulting in a pixel size of 1.014 Å/pixel. The non–dose-weighted images were used for CTF estimation by CTFfind 4.18 [41]. The dose-weighted images were used for particles picking; 191,921 particles were semi-automatically picked by Gautomatch (http://www.mrc-lmb.cam.ac.uk/kzhang/) and extracted by Relion-2.1 [42] in a box size of 220 pixels. Two-dimensional classification was performed in Relion-2.1 to remove contaminations, ice, and bad particles, yielding 110,752 good particles. The initial model was generated using the PDB file of the crystal structure by Molmap command in Chimera [43] and low-pass filtered to 10 A° resolution for three-dimensional classification in Relion-2.1. A round of three-dimensional classification into 6 classes yielded 1 reasonable reconstruction (Class 1, 22,442 particles) for further refinement. Refinement of the selected particles generated a map with an average resolution of 5.7 A° for the C3 symmetry. The map was sharpened with B-factors of −150 A°^2^. The ResMap [44] method was used to calculate the local resolution of our final dOrai-P288L map. All the figures were prepared in PyMol software (https://pymol.org/) and Chimera [43]. Data collection and reconstruction statistics are presented in S2 Text.

### Cell culture and transfection

HEK293T was cultured in DMEM (Sigma) supplemented with 10% fetal bovine serum (FBS, PAN) at 37°C with 5% CO_2_. Sf9 cells grown in Insect X-Press (Lonza, Basel, Switzerland) at 27°C. HEK293S GnTi^−^ was cultured in Freestyle 293 (Invitrogen, Carlsbad) at 37°C with 5% CO_2_. Bacmid was transfected into Sf9 cells using X-tremeGENE HP DNA Transfection Reagent (Roche, Indianapolis). All plasmids were transfected into HEK293T cells using Fugene6 (Promega, Madison).

### Whole-cell patch clamp recordings

HEK293T cells were transiently transfected with either GFP-tagged dOrai-P288L alone or hOrai1-GFP (wild type and mutants) plus human STIM1-YFP for 12 to 24 h at 37°C with 5% CO_2_. Transfected cells were then digested by trypsin to be plated onto 35-mm dishes for cultivation for at least 3 h before electrophysiology. The glass pipettes were pulled to a suitable shape through using a P-97 glass microelectrode puller (Sutter Instrument, Novato) and polished with an MF-830 (Narishige, Tokyo, Japan). For whole-cell configuration of recording dOrai-P288L currents, all internal pipette solutions are 160 mM NMDG, 10 mM HEPES, and 10 mM glucose (pH adjusted to 7.2 with HCl). The resistance was 3 to 5 MΩ after being filled with the internal recording solution. For whole-cell configuration of recording Ca^2+^ currents, the external bath Ca^2+^ solution was 105 mM CaCl_2_, 10 mM HEPES, and 10 mM glucose (pH adjusted to 7.4 with Trizma base). For whole-cell configuration of recording Na^+^ or K^+^ currents, the external bath Na^+^ or K^+^ solution was 160 mM NaCl or 160 mM KCl, 10 mM HEPES, and 10 mM glucose (pH adjusted to 7.4 with Trizma base). All experiments were conducted at room temperature with the stimulation voltage: 50 ms voltage step to −100 mV from a holding potential of 0 mV, followed by a voltage ramp increasing from −100 to +100 mV in 50 ms with a frequency of 0.5 Hz. In the whole-cell configuration, the 0 Ca^2+^, 0 Na^+^_,_ and 0 K^+^ external solutions were replaced, respectively, with the external Ca^2+^, Na^+^, and K^+^ solutions through a peristaltic pump to record the corresponding currents. For whole-cell configuration of recording hOrai1 currents, the internal pipette solutions are 135 mM L-Aspartic acid (or 100 mM D-Sorbitol with 75 mM L-Aspartic acid or 200 mM D-Sorbitol with 25 mM L-Aspartic acid or 250 mM D-Sorbitol only), 10 mM EGTA, 10 mM HEPES, and 8 mM MgCl_2_ (pH adjusted to 7.2 with CsOH). The extracellular 0 Ca^2+^ bath solution is 130 mM NaCl, 4.5 mM KCl, 22 mM MgCl_2_, 10 mM TEA-Cl, 10 mM D-glucose, and 5 mM Na-HEPES (pH 7.4). The extracellular Ca^2+^-containing bath solution is 130 mM NaCl, 4.5 mM KCl, 2 mM MgCl_2_, 20 mM CaCl_2_, 10 mM TEA-Cl, 10 mM D-glucose, and 5 mM Na-HEPES (pH 7.4). Whole-cell currents were amplified with an Axopatch 700B and digitized with a Digidata 1550A system (Molecular Devices, Sunnyvale). All currents were sampled at 10 kHz and low-pass filtered at 2 kHz through pCLAMP software (Molecular Devices, Sunnyvale). Origin 9.0 software (OriginLab Corp., Northampton) also was used for data analysis.

### Planar lipid bilayer formation

The planar lipid bilayer was formed on a Nanion NPC-1 chip from the giant unilamellar vesicles (GUVs) generated through the electroformation methods (Nanion, Munich, Germany). Materials required for GUV formation are listed as follows: Diphytanoylsn-glycero-3-phosphatidylcholine (DPhPC) lipids (Avanti Polar Lipids, Alabaster), cholesterol (Sigma, St. Louis), chloroform (Carl Roth, Karlsruhe, Germany), sorbitol (Sigma, St. Louis), NPC-1 chip with an aperture, and Vesicle Prep Pro (with electroformation chambers containing ITO slides, O-ring). DPhPC and cholesterol were dissolved in chloroform to 10 mM and 1 mM, respectively, and sorbitol was dissolved in double distilled water to 1 M. An electroformation protocol of 3 V peak to peak and 5 Hz frequency at 37°C lasting 188 min was used to increase the number of GUVs. GUVs were verified under the microscope.

### Single-channel recordings

The internal solution contained 50 mM CsCl, 10 mM NaCl, 60 mM CsF, 20 mM EGTA, and 10 mM HEPES (pH adjusted to 7.2 with CsOH; 285 mOsmol). The internal solution was first added onto both surfaces of the chip aperture. GUVs were then added and positioned onto the aperture by a slight negative pressure (planar lipid bilayers formation on the Port-a-Patch; Nanion, Munich, Germany). The GUV burst to form the planar lipid bilayer with a high seal resistance of about tens to hundreds of GΩ after touching the glass chip. The enhancer solution (Nanion, Munich, Germany) containing 80 mM NaCl, 3 mM KCl, 10 mM MgCl_2_, 35 mM CaCl_2_, and 10 mM HEPES (pH adjusted to 7.4 with NaOH; 298 mOsmol) was used as the external recording solution. Single-channel recordings were conducted using the PatchMaster software with a HEKA EPC10 USB amplifier (Heka Health, Westfield). Current signals were acquired using PatchMaster software with a 10-kHz sampling frequency and were filtered at 2 kHz. The holding potential was 0 mV. Stimulation potentials were given from the *cis* side of the glass chip chamber. Zero potential was assigned by convention to the *trans* side of the chip (the grounded side). Purified proteins were added to the external surface of the chip (the *trans* side). Acquired data were analyzed by using Origin 9.0. All experiments were performed at room temperature.

### Co-IP and western blotting

Cells were harvested after 24 h of plasmid transfection. Before collection, cells were washed 3 times with TBS and treated by ionomycin for 5 min at room temperature. Cells were lysed by buffer containing 20 mM Tris-HCl (pH 7.5), 150 mM NaCl, 0.5% Triton X-100, Roche protease inhibitor cocktail, and 1 mM EGTA at 4°C. After 1 h, cell supernatant was collected by centrifugation at 20,000*g* at 4°C for 30 min; 1 mL cell lysates were incubated with 15 μL GFP-Trap (ChromoTek) for 2 h at 4°C. The beads were washed 3 or 4 times by cold washing buffer composed of 20 mM Tris-HCl (pH 7.5), 150 mM NaCl, 0.1% Triton X-100, and 1 mM EGTA, followed by heating in 30 μL buffer containing SDS gel loading at 95°C for elution. Afterwards, eluted protein was loaded onto 11% SDS-PAGE gel and transferred to PVDF membranes for western blotting. The primary antibody of immunoblot analysis is anti-myc (M5546; Sigma-Aldrich, St. Louis) and anti-GFP (ab6556; Abcam, Cambridge, England). The protein–antibody complexes were detected using chemiluminescence.

### FRET measurements

FRET was measured as described earlier by Li and colleagues [45]. In brief, hOrai1-CFP (donor) and STIM1-YFP (acceptor) were transiently transfected into HEK293T cells. Imaging was measured utilizing a Leica DMI6000B microscope equipped with high-speed fluorescence-external filter wheels for a CFP-YFP index-based FRET experiment. Images were captured every 10 s using a 63× oil/1.4 oil objective (Leica) and controlled by LAS software. Data were analyzed by the Biosensor Processing Software 2.1 package in MATLAB R2014a (https://www.mathworks.com/products/matlab.html) and ImageJ (https://imagej.nih.gov/ij/). Bleed-through and direct excitation containment were subtracted according to the formula Fc = I_DA_ − aI_AA_−dI_DD_ (assuming *b* = *c* = 0), in which “d” represents bleed-through of CFP through the FRET filter (d = 0.715) and “a” represents direct excitation factor of YFP through the FRET filter (a = 0.173). The Eapp was analyzed using the equation Eapp = Fc ÷ (Fc + GI_DD_), in which Eapp represents the fraction of donor (ECFP) exhibiting FRET. The D1ER cameleon was used to determine the instrument-specific constant G (G = 3.458).

### Intracellular Ca^2+^ measurement

Intracellular Ca^2+^ measurement was performed on HEK-293T cells 20 to 24 h after transfection with STIM1-YFP and hOrai1-GFP. Cells were treated with 2 μM fura-2/AM (Sigma, St. Louis) in standard Ringer’s solution (140 mM NaCl, 10 mM HEPES [pH 7.4], 10 mM Glucose, 0.8 mM MgCl_2_, 2.8 mM KCl, 2 mM CaCl_2_) for 30 min and then were re-incubated in fresh Ringer’s solution for another 30 min. All these processes were done at room temperature. Fluorescence imaging was measured using a Leica DMI6000B microscope with a 40× oil-immersion objective lens controlled by LAS software (https://www.leica-microsystems.com/products/microscope-software/). Consecutive excitation occurred at 340 and 380 nm, and emission was collected at 510 nm. Intracellular Ca^2+^ concentration is shown as the 340/380 ratio (F340/F380) obtained from 16 to 41 cells.

## Supporting information

S1 Fig Sequence alignment. The residues that are conserved among all 4 proteins are highlighted in red. The accession numbers for the sequences in the alignment are Q9U6B8 for fly Orai, Q96D31 for hOrai1, Q96SN7 for hOrai2, and Q9BRQ5 for hOrai3, respectively. The star symbol denotes the amino acids mutated in structural studies. hOrai, human Orai.
(TIF)

S2 Fig Biochemical properties of the dOrai-P288L channel. (A) Fluorescence microscopy picture of the dOrai-P288L channel localized at the surface of HEK-293T cells. (B) Gel-filtration profile of the purified dOrai-P288L channel. The inset shows the purity of the dOrai-P288L channel observed by SDS-PAGE. Primary data can be found in S1 Data. dOrai, *Drosophila melanogaster* Orai; HEK, human embryonic kidney.
(TIF)

S3 Fig Molecule packing in 1 asymmetric unit of dOrai-P288L crystal. Two hexamers of the dOrai-P288L channel are shown in blue and red. The electron density is drawn in gray. dOrai, *Drosophila melanogaster* Orai.
(TIF)

S4 Fig Cryo-EM structure determination and resolution assessment of the dOrai-P288L channel. (A) A drift-corrected cryo-EM micrograph of the dOrai-P288L channel. (B) Ctffind showed Thon rings in the Fourier spectrum of the image in panel A. (C) Selected two-dimensional class averages of the dOrai-P288L channel. (D) The gold-standard FSC coefficient curve of the final reconstruction showed an overall resolution of 5.7 Å. (E) Local resolution estimation by ResMap (http://resmap.sourceforge.net/). cryo-EM, cryo-electron microscopy; dOrai, *Drosophila melanogaster* Orai; FSC, Fourier shell correlation.
(TIF)

S5 Fig Cryo-EM data processing of dOrai-P288L channel. (A) Flow chart of whole-data processing. (B) Orientation distribution of particles included in the final reconstruction. cryo-EM, cryo-electron microscopy; dOrai, *Drosophila melanogaster* Orai.
(TIF)

S6 Fig Bottom view of the overlay of the crystal structure (color cartoon) of the closed dOrai channel and the cryo-EM map (white surface) of the dOrai-P288L channel. The crystal structure cannot be fitted into the cryo-EM map. cryo-EM, cryo-electron microscopy; dOrai, *Drosophila melanogaster* Orai.
(TIF)

S7 Fig Conformational changes between the closed and open dOrai channels. The closed dOrai channel is colored gray, and the open dOrai channel is colored orange. Two opposing protomers are shown. Side chains of residues K163, K159, and R155 are shown as stick models. The amino acid numbers of hOrai1 are shown in parentheses. cryo-EM, cryo-electron microscopy; dOrai, *Drosophila melanogaster* Orai; hOrai, human Orai.
(TIF)

S8 Fig TM3–TM4 hydrophobic interaction is essential for STIM1-dependent hOrai1 activation. (A) Bar graphs of whole-cell Ca^2+^ currents of STIM1-activated wild-type and mutant hOrai1 channels (hOrai1, hOrai1-L261A, and hOrai1-F257A). (B) Extracellular Ca^2+^ influx in HEK-293T cells co-expressing STIM1-YFP and wild-type or mutant hOrai1-GFP. (C) FRET between STIM1-YFP (acceptor) and wild-type or mutant hOrai1-CFP (donor) co-expressed in HEK-293T cells. The curves of F257A and L261A are colored red and blue, respectively. The number of analyzed cells is indicated. Error bars denote SEM. ***p < 0.001 (unpaired Student *t* test). Primary data can be found in S1 Data. FRET, fluorescence resonance energy transfer; GFP, green fluorescent protein; hOrai, human Orai; STIM1, stromal interaction molecule; TM, transmembrane; YFP, yellow fluorescent protein.
(TIF)

S9 Fig Interactions between the TM1 helix and the TM3 helix in the closed dOrai structure. (A) The TM1 helix and the TM3 helix are colored green and orange, respectively. The ion-conducting pore side is labeled. (B) Zoom view of the specific interactions between 2 helices. Side chains of 3 residues (K157, S154, and L153) from the TM1 helix, and 2 residues (E245 and H241) from the TM3 helix are shown. The hydrogen bonds are shown as magenta dashed lines. Atoms oxygen and nitrogen are colored red and blue, respectively. Amino acids in parentheses denote hOrai1 counterparts. The atom coordinates were taken from the structure with the RCSB code 4HKR. Side chains of residues K157 and L153 were absent in original PDB file. They were manually built from the program Coot (http://www2.mrc-lmb.cam.ac.uk/Personal/pemsley/coot/) based on frequently used rotamers. dOrai, *Drosophila melanogaster* Orai; hOrai, human Orai; PDB, Protein Data Bank; RCSB, Research Collaboratory For Structural Bioinformatics; TM, transmembrane.
(TIF)

S10 Fig Anions near the pore on the cytosolic side are critical for Ca^2+^ permeation. (A) Bar graphs of whole-cell Ca^2+^ currents of wild-type and mutant STIM1-activated hOrai1 channels (hOrai1, hOrai1-R83A-K87A, and hOai1-R77A-K78A). (B) Western blot analysis of coimmunoprecipitated hOrai1-GFP (wild type and mutants) with STIM1-myc. (C) Bar graphs of whole-cell Ca^2+^ currents of wild-type STIM1-activated hOrai1 channels with cesium aspartate at concentrations of 0, 25 mM, 75 mM, and 135 mM in the pipette solution. The number of analyzed cells is indicated. *p < 0.05; ***p < 0.001 (unpaired Student *t* test). Error bars denote SEM. Primary data can be found in S1 Data. co-IP, coimmunoprecipitation; GFP, green fluorescent protein; hOrai, human Orai; STIM1, stromal interaction molecule.
(TIF)

S1 Text Crystallographic data collection and refinement statistics. (DOCX)

S2 Text Cryo-EM data collection and reconstruction statistics. cryo-EM, cryo-electron microscopy.
(DOCX)

S1 Data Primary data set. This file contains individual numerical values used to generate figures in this manuscript.
(XLSX)

## Abbreviations

CFP: cyan fluorescent protein
CHS: cholesteryl hemisuccinate
co-IP: coimmunoprecipitation
cryo-EM: cryo-electron microscopyDDM, n-dodecyl-β-d-maltopyranoside
dOrai: *Drosophila melanogaster* Orai
DPhPC: Diphytanoylsn-glycero-3-phosphatidylcholine
Eapp: apparent FRET efficiency
eGFP: enhanced green fluorescent protein
ER: endoplasmic reticulum
FBS: fetal bovine serum
FRET: fluorescence resonance energy transfer
GDN: glyco-diosgenin
GFP: green fluorescent protein
GSK: GlaxoSmithKline
GUV: giant unilamellar vesicle
HEK: human embryonic kidney
hOrai: human Orai
NG: n-Nonyl-β-D-glucopyranoside
NM: n-Nonyl-β-D-Maltoside
PM: plasma membrane
RCSB: Research Collaboratory For Structural Bioinformatics
Sf9: *Spodoptera frugiperda 9*
SOC: store-operated calcium channel
SOCE: store-operated calcium entry
STIM: stromal interaction molecule
TM: transmembrane
YFP: yellow fluorescent protein
