# Supplementary figures and images for: Molecular understanding of calcium permeation through the open Orai channel
Source: PLoS Biol. 2019 Apr 22;17(4):e3000096. doi: 10.1371/journal.pbio.3000096 (PMC6497303; doi:10.1371/journal.pbio.3000096)

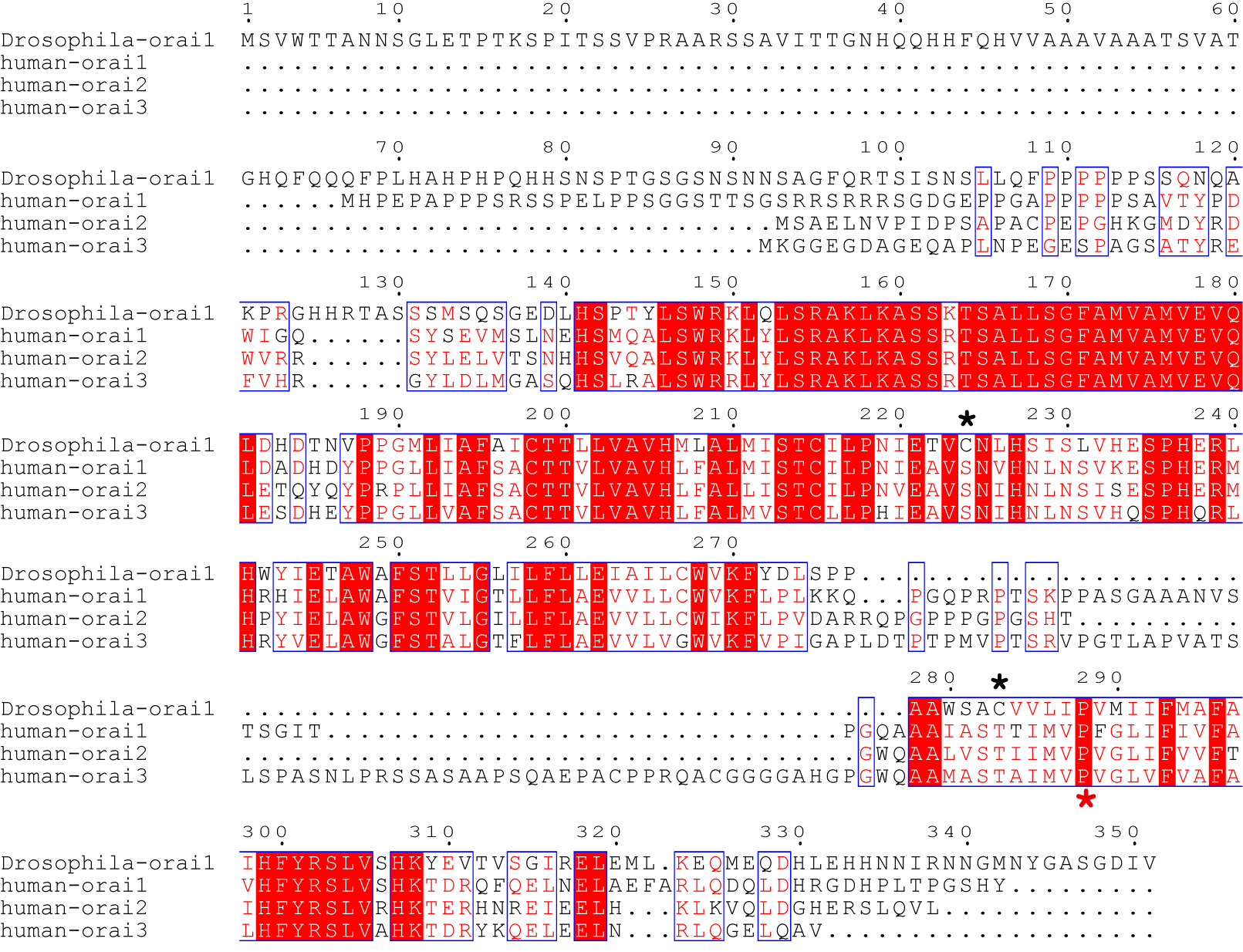

Supplement: S1 Fig — The residues that are conserved among all 4 proteins are highlighted in red. The accession numbers for the sequences in the alignment are Q9U6B8 for fly Orai, Q96D31 for hOrai1, Q96SN7 for hOrai2, and Q9BRQ5 for hOrai3, respectively. The star symbol denotes the amino acids mutated in structural studies. hOrai, human Orai. (TIF) [file pbio.3000096.s001.tif]

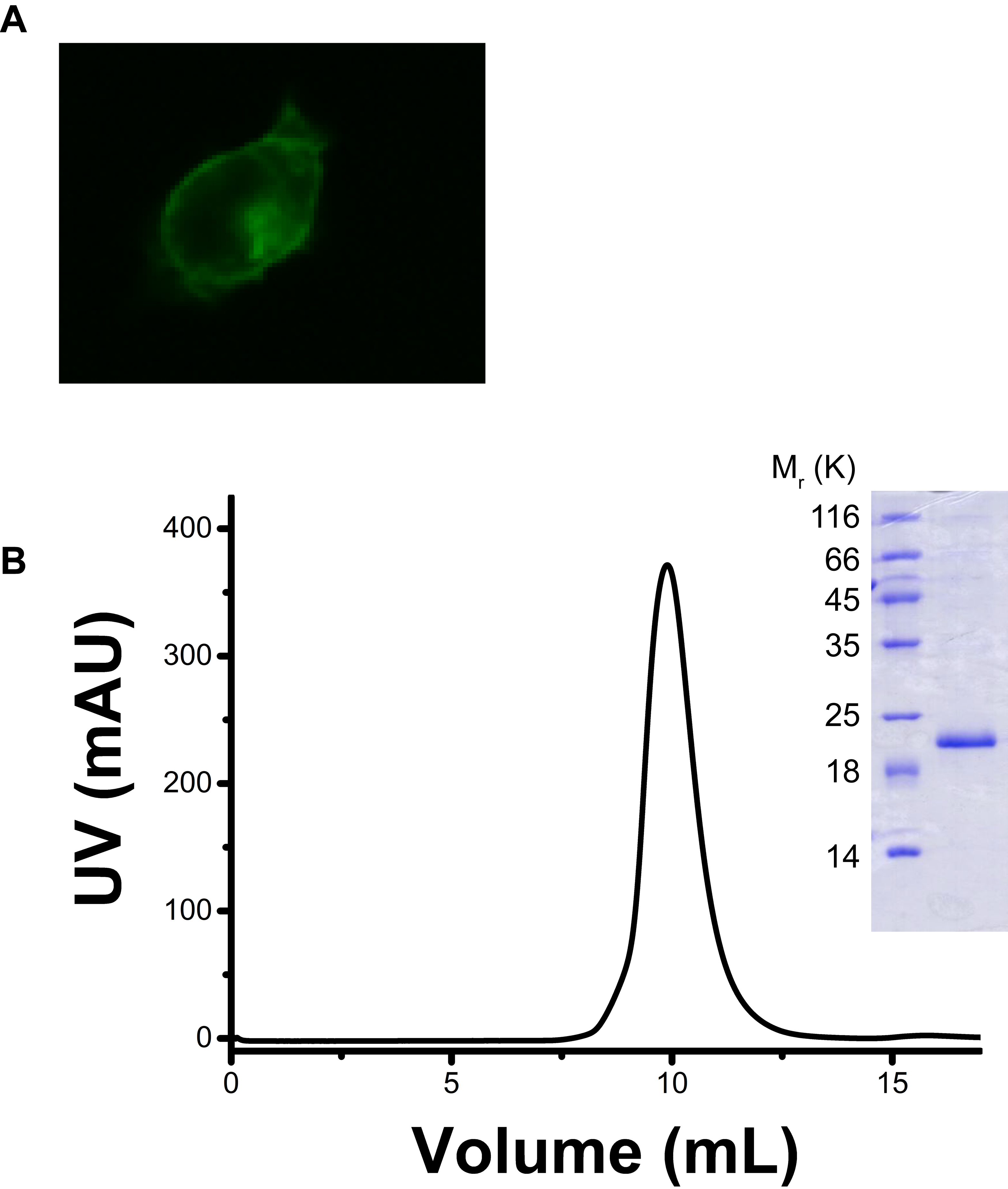

Supplement: S2 Fig — (A) Fluorescence microscopy picture of the dOrai-P288L channel localized at the surface of HEK-293T cells. (B) Gel-filtration profile of the purified dOrai-P288L channel. The inset shows the purity of the dOrai-P288L channel observed by SDS-PAGE. Primary data can be found in S1 Data. dOrai, Drosophila melanogaster Orai; HEK, human embryonic kidney. (TIF) [file pbio.3000096.s002.tif]

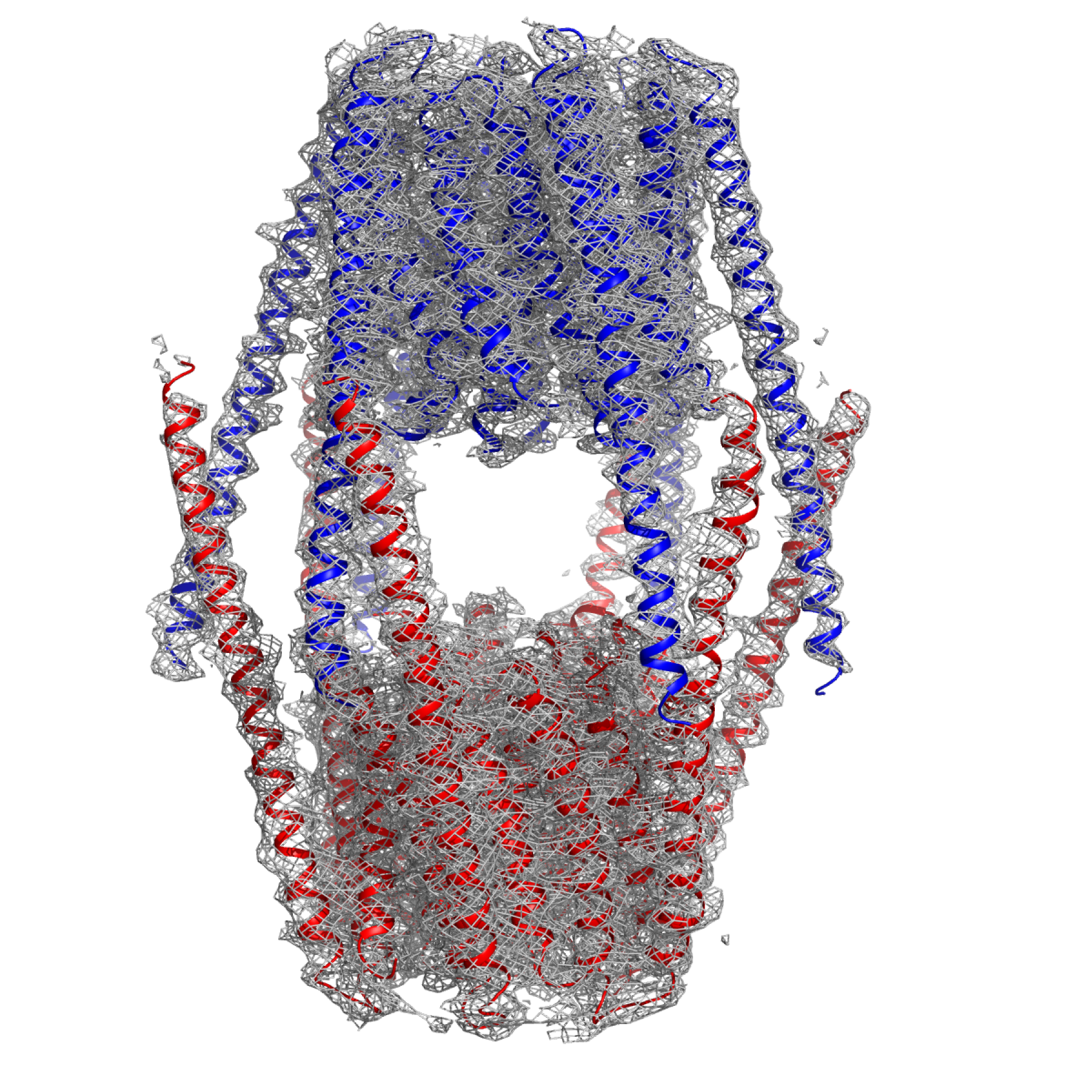

Supplement: S3 Fig — Two hexamers of the dOrai-P288L channel are shown in blue and red. The electron density is drawn in gray. dOrai, Drosophila melanogaster Orai. (TIF) [file pbio.3000096.s003.tif]

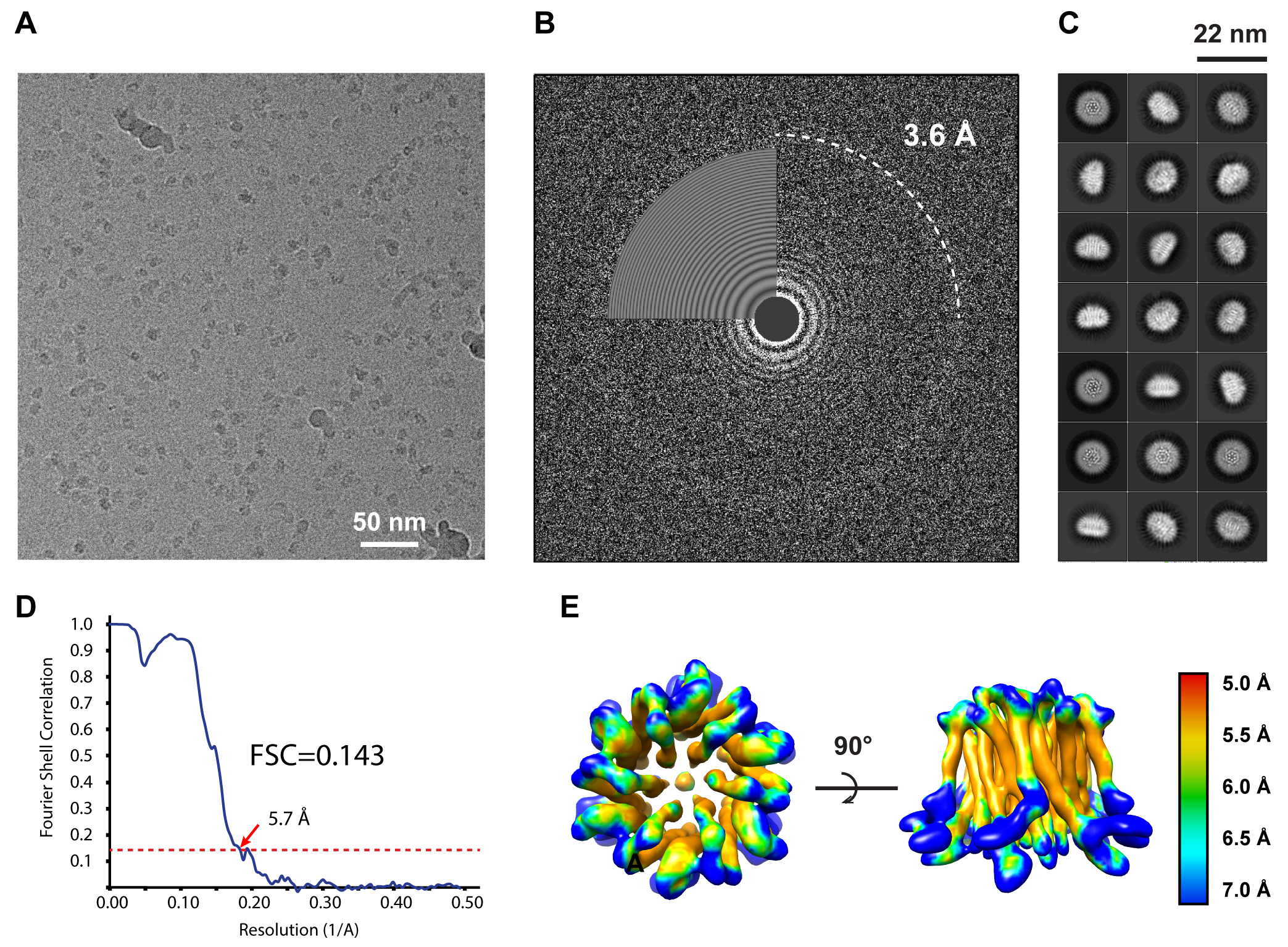

Supplement: S4 Fig — (A) A drift-corrected cryo-EM micrograph of the dOrai-P288L channel. (B) Ctffind showed Thon rings in the Fourier spectrum of the image in panel A. (C) Selected two-dimensional class averages of the dOrai-P288L channel. (D) The gold-standard FSC coefficient curve of the final reconstruction showed an overall resolution of 5.7 Å. (E) Local resolution estimation by ResMap (http://resmap.sourceforge.net/). cryo-EM, cryo-electron microscopy; dOrai, Drosophila melanogaster Orai; FSC, Fourier shell correlation. (TIF) [file pbio.3000096.s004.tif]

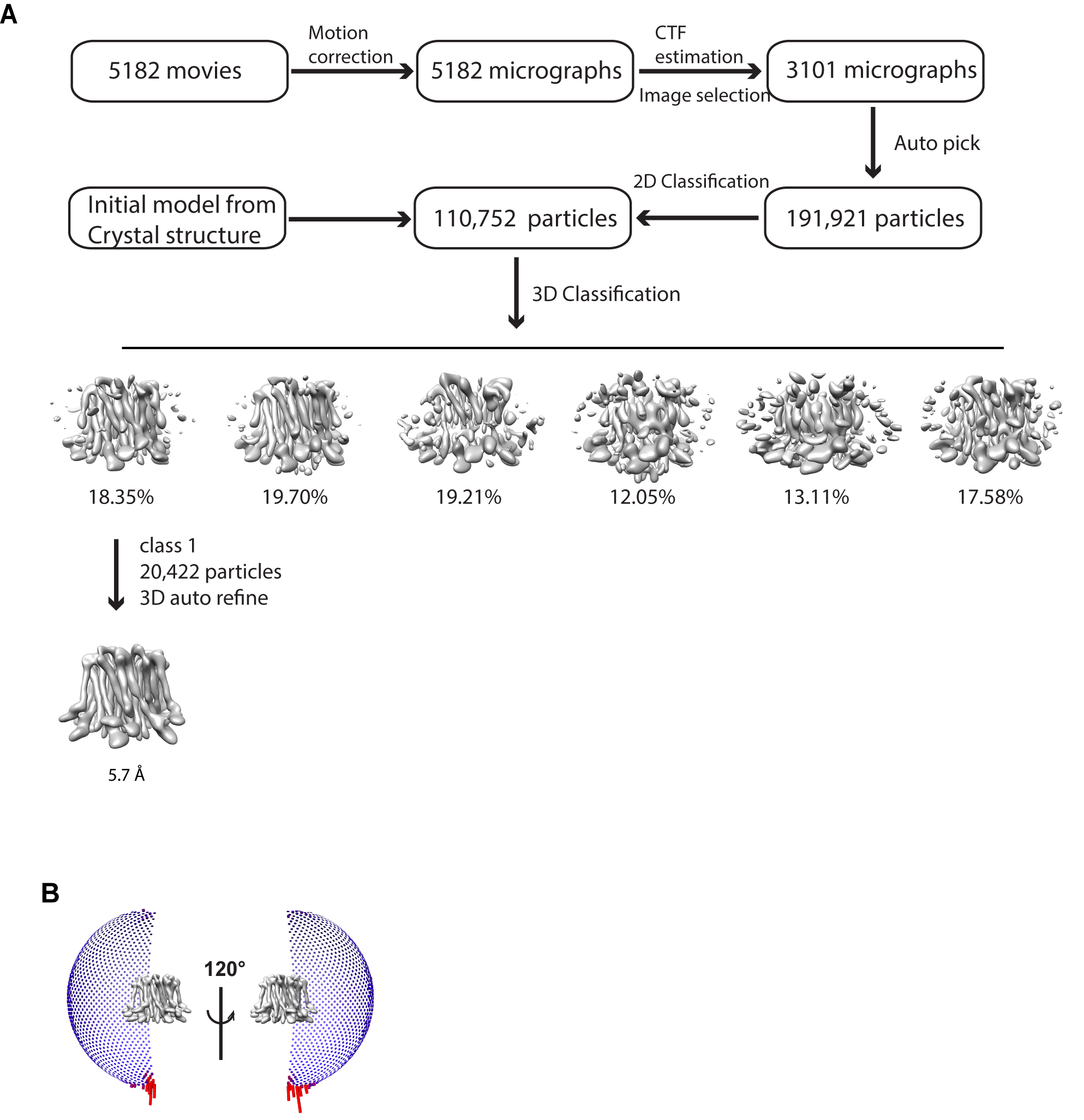

Supplement: S5 Fig — (A) Flow chart of whole-data processing. (B) Orientation distribution of particles included in the final reconstruction. cryo-EM, cryo-electron microscopy; dOrai, Drosophila melanogaster Orai. (TIF) [file pbio.3000096.s005.tif]

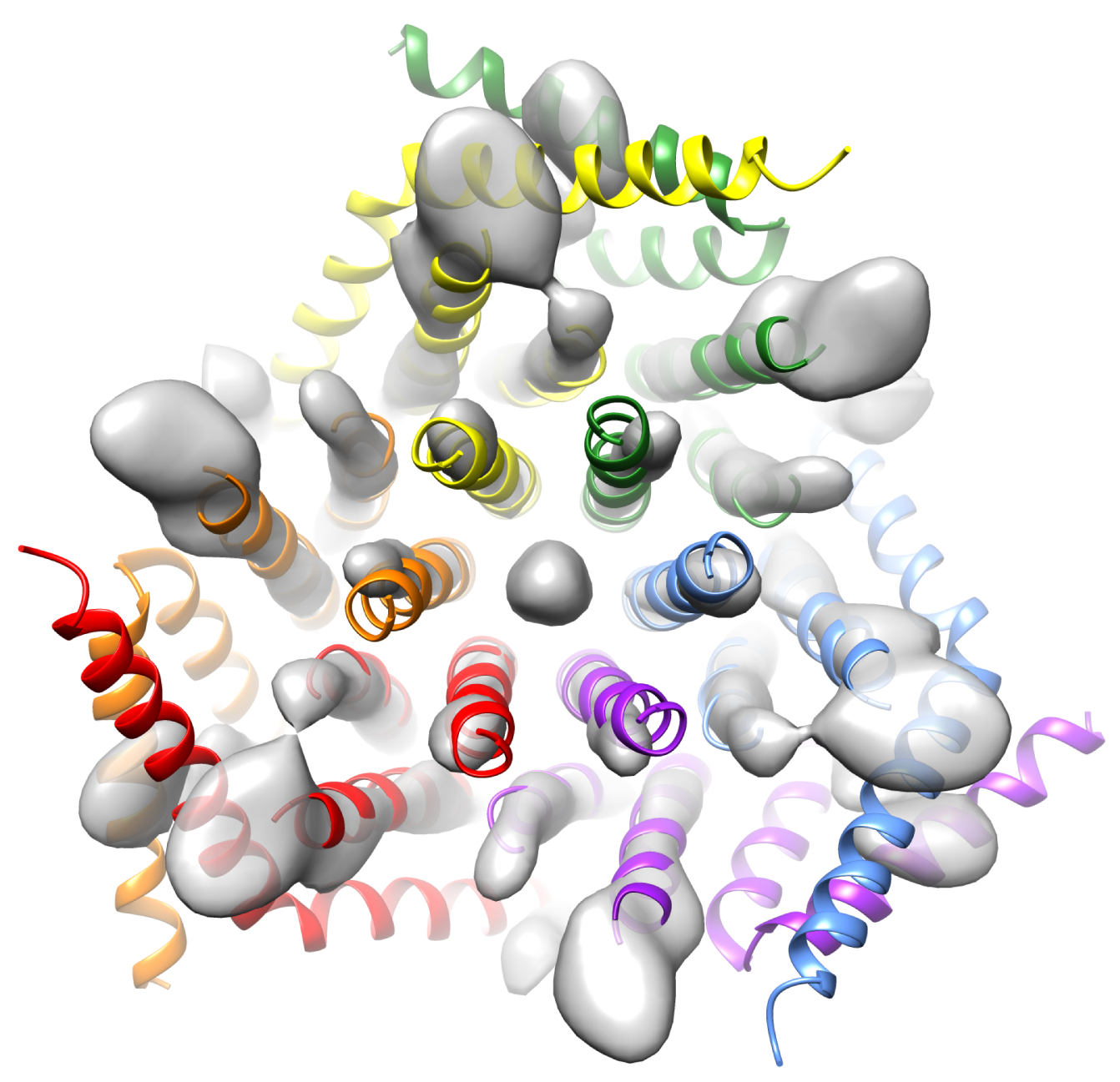

Supplement: S6 Fig — The crystal structure cannot be fitted into the cryo-EM map. cryo-EM, cryo-electron microscopy; dOrai, Drosophila melanogaster Orai. (TIF) [file pbio.3000096.s006.tif]

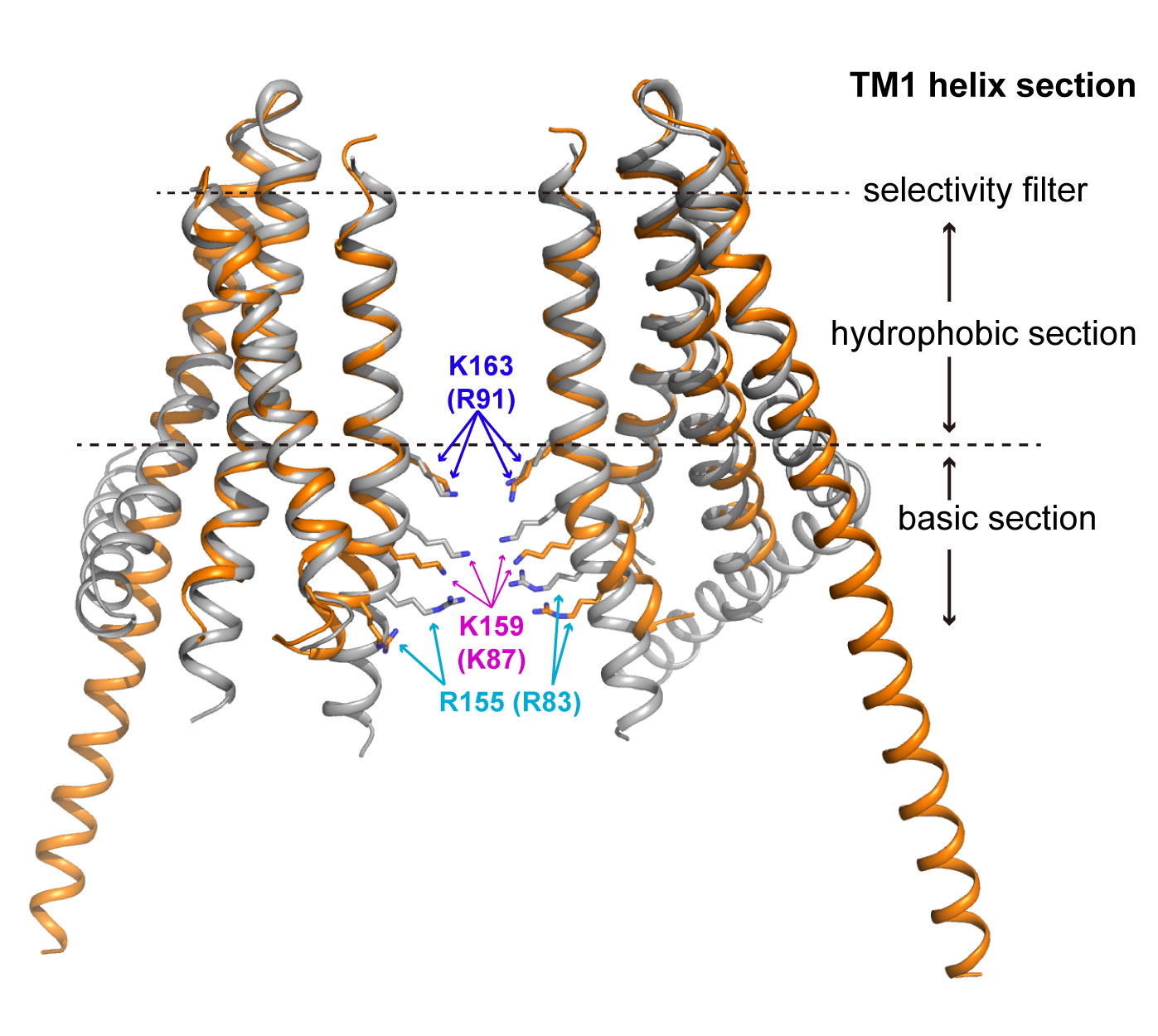

Supplement: S7 Fig — The closed dOrai channel is colored gray, and the open dOrai channel is colored orange. Two opposing protomers are shown. Side chains of residues K163, K159, and R155 are shown as stick models. The amino acid numbers of hOrai1 are shown in parentheses. cryo-EM, cryo-electron microscopy; dOrai, Drosophila melanogaster Orai; hOrai, human Orai. (TIF) [file pbio.3000096.s007.tif]

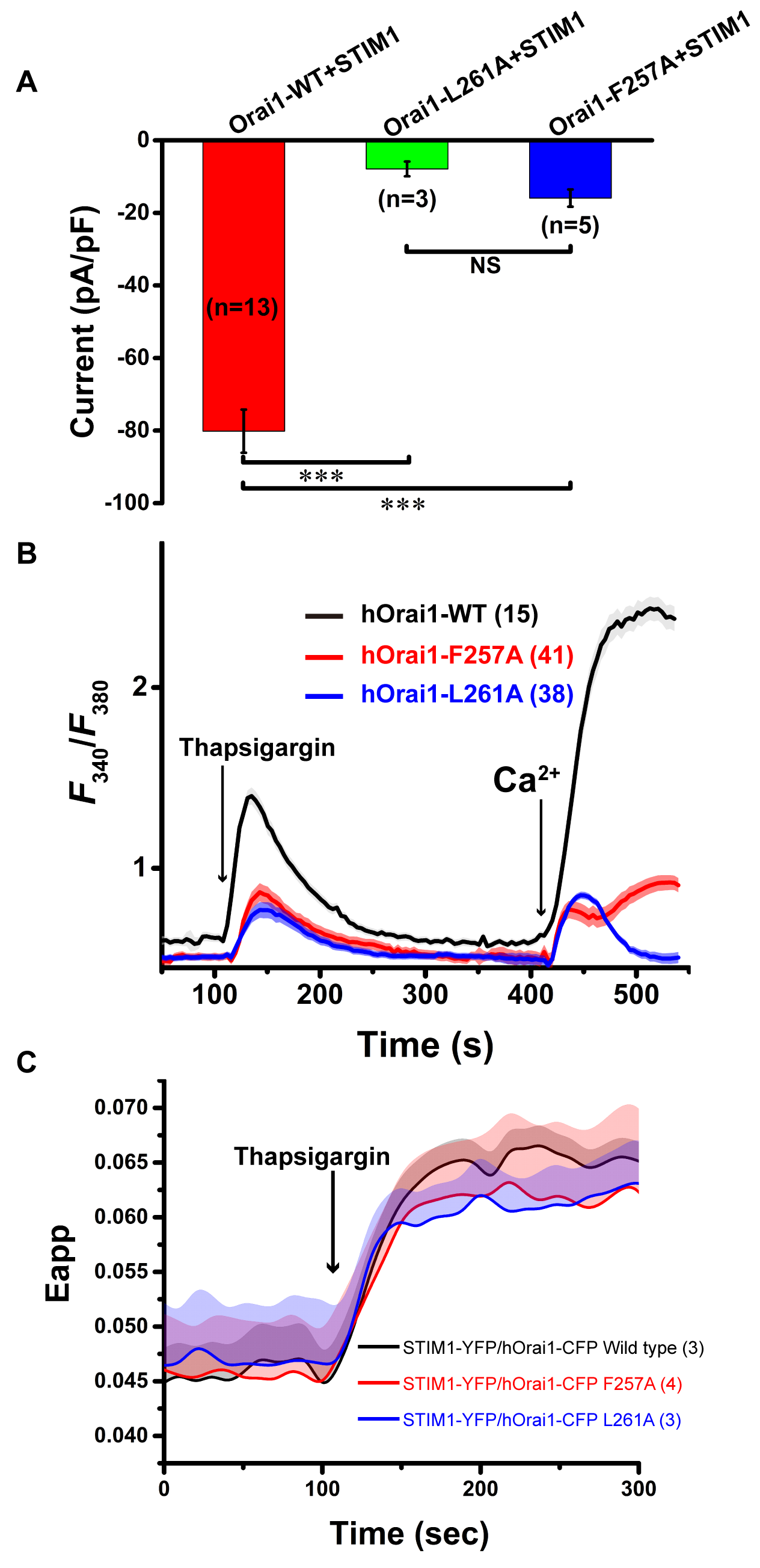

Supplement: S8 Fig — (A) Bar graphs of whole-cell Ca2+ currents of STIM1-activated wild-type and mutant hOrai1 channels (hOrai1, hOrai1-L261A, and hOrai1-F257A). (B) Extracellular Ca2+ influx in HEK-293T cells co-expressing STIM1-YFP and wild-type or mutant hOrai1-GFP. (C) FRET between STIM1-YFP (acceptor) and wild-type or mutant hOrai1-CFP (donor) co-expressed in HEK-293T cells. The curves of F257A and L261A are colored red and blue, respectively. The number of analyzed cells is indicated. Error bars denote SEM. ***p < 0.001 (unpaired Student t test). Primary data can be found in S1 Data. FRET, fluorescence resonance energy transfer; GFP, green fluorescent protein; hOrai, human Orai; STIM1, stromal interaction molecule; TM, transmembrane; YFP, yellow fluorescent protein. (TIF) [file pbio.3000096.s008.tif]

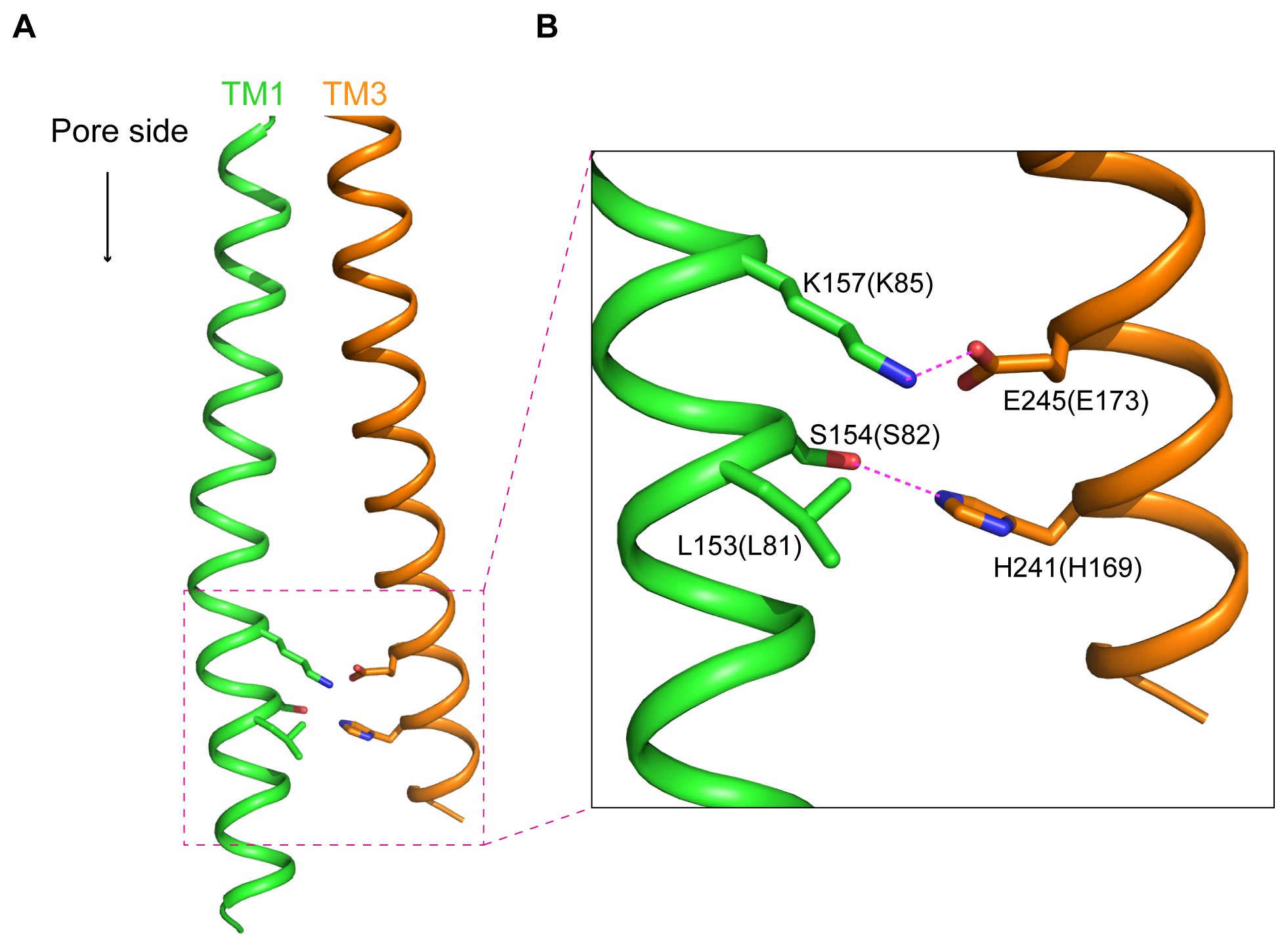

Supplement: S9 Fig — (A) The TM1 helix and the TM3 helix are colored green and orange, respectively. The ion-conducting pore side is labeled. (B) Zoom view of the specific interactions between 2 helices. Side chains of 3 residues (K157, S154, and L153) from the TM1 helix, and 2 residues (E245 and H241) from the TM3 helix are shown. The hydrogen bonds are shown as magenta dashed lines. Atoms oxygen and nitrogen are colored red and blue, respectively. Amino acids in parentheses denote hOrai1 counterparts. The atom coordinates were taken from the structure with the RCSB code 4HKR. Side chains of residues K157 and L153 were absent in original PDB file. They were manually built from the program Coot (http://www2.mrc-lmb.cam.ac.uk/Personal/pemsley/coot/) based on frequently used rotamers. dOrai, Drosophila melanogaster Orai; hOrai, human Orai; PDB, Protein Data Bank; RCSB, Research Collaboratory For Structural Bioinformatics; TM, transmembrane. (TIF) [file pbio.3000096.s009.tif]

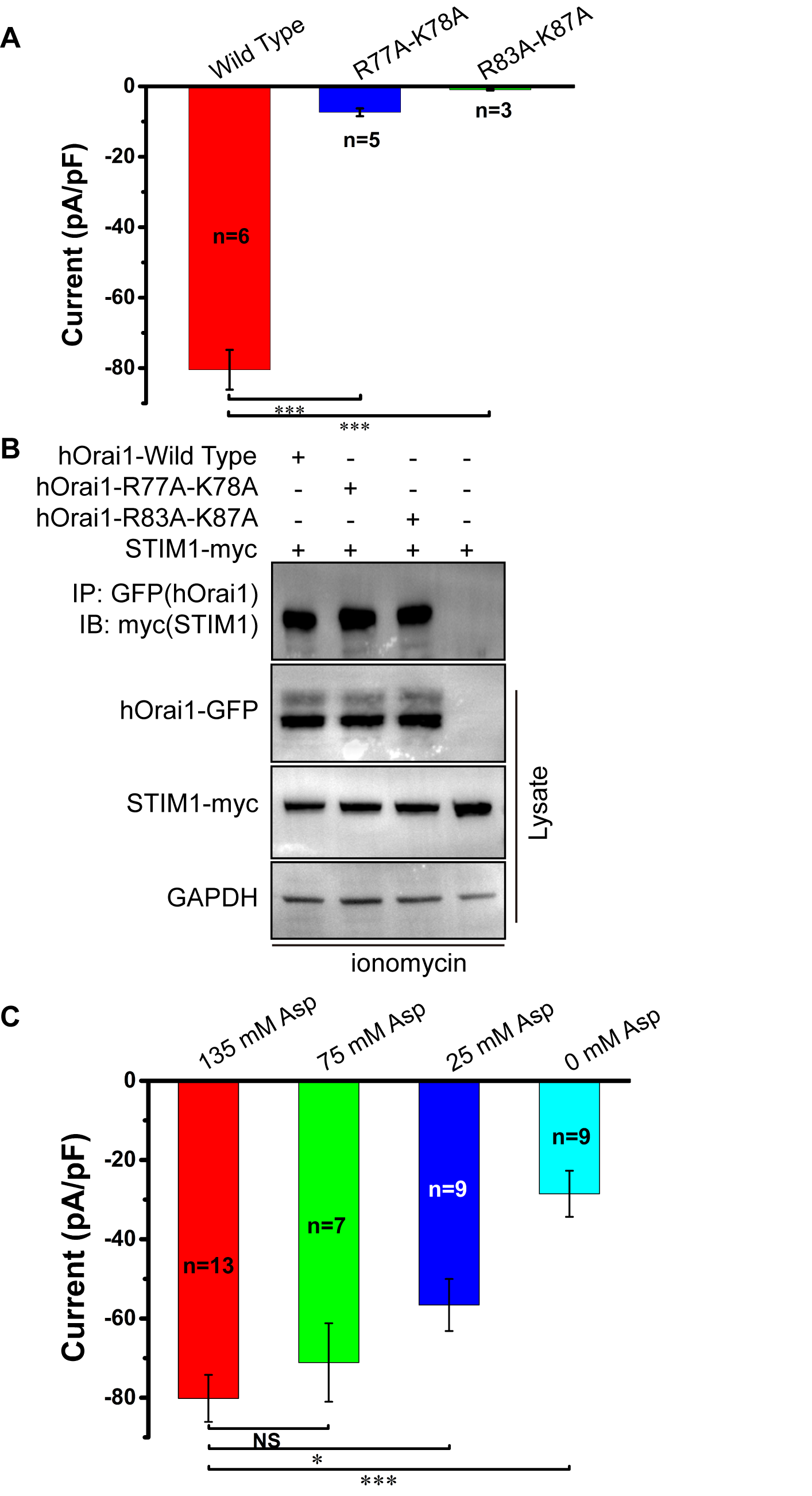

Supplement: S10 Fig — (A) Bar graphs of whole-cell Ca2+ currents of wild-type and mutant STIM1-activated hOrai1 channels (hOrai1, hOrai1-R83A-K87A, and hOai1-R77A-K78A). (B) Western blot analysis of coimmunoprecipitated hOrai1-GFP (wild type and mutants) with STIM1-myc. (C) Bar graphs of whole-cell Ca2+ currents of wild-type STIM1-activated hOrai1 channels with cesium aspartate at concentrations of 0, 25 mM, 75 mM, and 135 mM in the pipette solution. The number of analyzed cells is indicated. *p < 0.05; ***p < 0.001 (unpaired Student t test). Error bars denote SEM. Primary data can be found in S1 Data. co-IP, coimmunoprecipitation; GFP, green fluorescent protein; hOrai, human Orai; STIM1, stromal interaction molecule. (TIF) [file pbio.3000096.s010.tif]
