# Supplementary material for: Molecular understanding of calcium permeation through the open Orai channel
Source: PLoS Biol. 2019 Apr 22;17(4):e3000096. doi: 10.1371/journal.pbio.3000096 (PMC6497303; doi:10.1371/journal.pbio.3000096)
Supplement: S1 Text — (DOCX) [file pbio.3000096.s011.docx]

**S1 Text. Crystallographic data collection and refinement statistics**

| Crystal name | **dOrai-P288L** |
| --- | --- |
| Space group | ***P2*_1_** |
| Unit cell |  |
| ***a, b, c*** (Å) | ***a****=*118.107*,* ***b****=*90.659, ***c****=*178.721 |
| *α, β, γ* (°) | *α=γ=*90, *β=*106.31 |
| Wavelength (Å) | 0.9800 |
| Resolution range (Å) | 50-4.50 (4.66-4.50)^**^ |
| No. of unique reflections | 17,659 (850)^**^ |
| Redundancy | 3.5 (2.7)^**^ |
| ***R***_sym_ (%)* | 12.4 (58.7)^**^ |
| ***I***/***σ*** | 7.94 (2.00)^**^ |
| Completeness (%) | 80.7 (39.8)^**^ |
| Figure of merit |  |
| Refinement |  |
| ***R***_free_ (%)^†^ | 38.83 |
| ***R***_crystal_ (%)^‡^ | 32.57 |
| RMSD_bond_ (Å) | 0.004 |
| RMSD_angle_(°) | 0.93 |
| Number of |  |
| Protein atoms | 12,372 |
| Ligand atoms | 4 |
| Solvent atoms | 0 |
| Residues in (%) |  |
| Ramachandran favored | 95.08 |
| Ramachandran allowed | 4.57 |
| Ramachandran outliers | 0.17 |
| Rotemer outliers | 0 |
| Average B factor (Å^2^) of |  |
| Protein | 122.88 |
| Ligand | 87.74 |
| Solvent | - |

****R***_sym_ = $\sum_{j} |I_{j}-\left\langle I\right\rangle|/\sum\left\langle I\right\rangle$ where $I_{j}$ is the intensity of the jth reflection and $\left\langle I\right\rangle$ is the average intensity.

** the highest resolution shell.

†***R***_crystal_ = $\sum_{hkl} ||F_{obs}|-|F_{calc}||/\sum_{hkl} |F_{obs}|$.

‡***R***_free_, calculated the same as ***R***_crystal_, but from a test set containing 5% of data excluded from the refinement calculation.
