# Supplementary material for: Molecular understanding of calcium permeation through the open Orai channel
Source: PLoS Biol. 2019 Apr 22;17(4):e3000096. doi: 10.1371/journal.pbio.3000096 (PMC6497303; doi:10.1371/journal.pbio.3000096)
Supplement: S2 Text — cryo-EM, cryo-electron microscopy. (DOCX) [file pbio.3000096.s012.docx]

**S2 Text. CryoEM data collection and Reconstruction statistics**

| **Data Collection** |  |
| --- | --- |
| EM equipment | FEI Titan Krios |
| Voltage (KV) | 300 |
| Detector | Gatan K2 |
| Pixel size (Å) | 1.014 |
| Electron Dose (e^-^/Å^2^) | 49.8 |
| Defocus range (μm) | -2.5~-1.5 |
| **Reconstruction** |  |
| Software | Relion 2.1 |
| Number of used particles | 20,422 |
| Accuracy of rotation (°) | 3.675 |
| Accuracy of translation (pixels) | 1.357 |
| Map sharpening B-factors (Å^2^) | -150 |
| Final Resolution (Å) | 5.7 |
